# Cost‐effectiveness of Resonance® metallic ureteral stent compared with standard polyurethane ureteral stents in malignant ureteric obstruction: A cost‐utility analysis

**DOI:** 10.1002/bco2.332

**Published:** 2024-03-20

**Authors:** Dawn M. Cooper, Rachel Lines, Iqbal Shergill

**Affiliations:** ^1^ Cook Medical Altrincham UK; ^2^ The Alan de Bolla Wrexham Urology Unit Wrexham Maelor Hospital Wrexham UK; ^3^ Maelor Academic Unit of Medical and Surgical Sciences Wrexham Maelor Hospital Wrexham UK

**Keywords:** cost‐effectiveness, double‐J stents, malignant ureteric obstruction, metallic stents

## Abstract

**Background:**

Malignant ureteral obstruction (MUO) is a frequent challenge for urologists. Patients have poor prognoses, treatment aims to improve quality‐of‐life while optimising renal function. Standard practice in the United Kingdom is to use polyurethane stents, which require frequent surgical replacements for blockages and encrustation. More durable metallic stents are available, although these incur an increased initial purchase price.

**Aims:**

We aim to assess whether the use of polyurethane double‐J (JJ) or metallic stent, Resonance® is more cost‐effective for managing MUO in the UK healthcare setting.

**Methods:**

A Markov model was parameterised to 5 years with costs and health‐related quality‐of‐life consequences for treating MUO with Resonance metallic stent (Cook Medical), versus standard JJ stents, from the UK care system perspective, with 3.5% discounting. Deterministic and probabilistic sensitivity analyses were undertaken to assess the effect of uncertainty.

**Results:**

Over 5 years, approximately four fewer repeat surgical interventions were estimated in the metallic stent arm compared with the JJ stent, driving a 23.4% reduction in costs. The mean estimates of costs and benefits indicate that treatment of MUO with Resonance for 5 years is dominant over JJ stents. Over 5 years a cost‐saving of £2164.74 and a health gain of +0.046 quality‐adjusted life years (QALYs) per patient is estimated. With a maximum willingness to pay of £20 k per QALY, a net monetary benefit (NMB) of £3077.83 is estimated. Probabilistic sensitivity analysis at a willingness to pay threshold of £20 000 indicates an 89.3% probability of Resonance being cost‐effective over JJ stents. Within 1‐year savings of £726.53 are estimated driven by a reduction of two fewer repeat surgical interventions when using the metallic stent.

**Conclusions:**

Resonance metallic stents for the treatment of MUO reduce the number of repeat procedures and could be a cost‐effective option for the treatment, potentially offering efficiencies to the healthcare system.

## 1 INTRODUCTION

Malignant ureteral obstruction (MUO), a frequent cause of obstructive uropathy, is often caused by extrinsic compression and can be difficult to manage. If left untreated, MUO can be deleterious to the patient's overall health, leading to renal failure and preventing systemic chemotherapies.^1^, ^2^ Given the poor prognosis, management of MUO aims to relieve symptoms and optimise renal function to facilitate chemotherapy treatments, while minimising hospitalisation and negative impact on quality‐of‐life.^1^, ^3^

Surgical reconstruction of the ureter is curative, but the invasiveness of such procedures makes them unsuitable for palliative care. Given this, MUO is generally treated with either a ureteric stent or percutaneous nephrostomy (PCN).^1^ Although PCN is frequently successful, due to the discomfort and inconvenience of external drainage, patients prefer ureteric stents.^2^, ^4^ Traditional JJ stents are effective in propping open the obstruction but require frequent changes; every 3–6 months,^5^ or sooner in case of failure.^5^, ^6^ Stent failure negatively impacts the patient's quality‐of‐life by worsening flank pain, deteriorating renal function, and worsening hydronephrosis. Furthermore, stent exchange can be technically difficult, may fail, or cause complications with added morbidity and thus further compromise the patient's quality of life.^1^, ^4^

A solution to frequent stent exchanges is metallic stents, designed to resist encrustation and external compressive forces, which are more effective in maintaining long‐term lumen patency.^7^ A potential limitation to the widespread use of metallic stents is the increased initial purchase price of the device compared with JJ stents. The economic impact of using metallic stents over JJ stents has been partially explored, but there is a lack of robust economic evidence, particularly for the MUO cohort.

### 1.1 Objective

This work aims to assess whether the use of the Resonance® metallic ureteral stent (Cook Medical) is more cost‐effective for managing MUO than JJ in the UK healthcare setting.

## 2 METHODS

### 2.1 Markov Model

**FIGURE 1:**
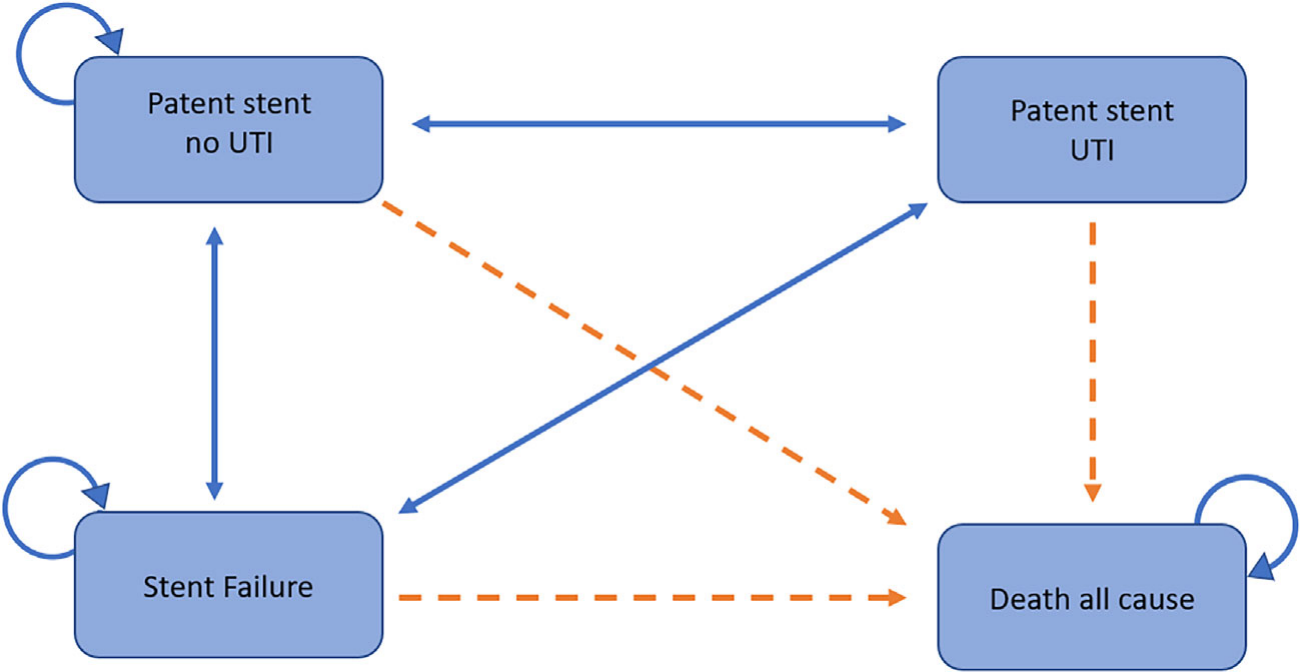
Markov model to assess the cost‐effectiveness of Resonance for malignant ureteric obstruction. UTI, urinary tract infection.

A Markov model was developed in Microsoft® Excel® 2013 to estimate the overall costs and consequences of treating MUO, with either JJ (standard practice in National Health Service ‐ NHS)^8^ or Resonance stents (a metallic, double pigtail stent). An NHS provider perspective was adopted with a discount rate of 3.5% applied to both costs and benefits. Consistent with MUO patients' expected lifetime survival, a 5‐year time horizon was adopted with monthly cycles used and half‐cycle correction applied. The results were reported in terms of total cost per patient treated, and outcomes were valued in terms of life years (LYs) and quality adjusted life years (QALYs) gained. The model began with a hypothetical cohort of MUO patients (aged 57–61, 33% male), treated with either Resonance or JJ stent. The individuals moved through one of four mutually exclusive health states: Patent stent, Patent stent with urinary tract infection, failed stent and death as depicted in Figure 1. The model assumed that UTIs resolved in one cycle and either continued to be patent or failed, a failed stent was replaced with another stent of the same type, death is an absorbing state and planned stent changes at 12 and 6 months were included for Resonance and JJ, respectively, in accordance with the instructions for use and standard practice in the United Kingdom.^8^

### 2.2 Literature Search

**FIGURE 2:**
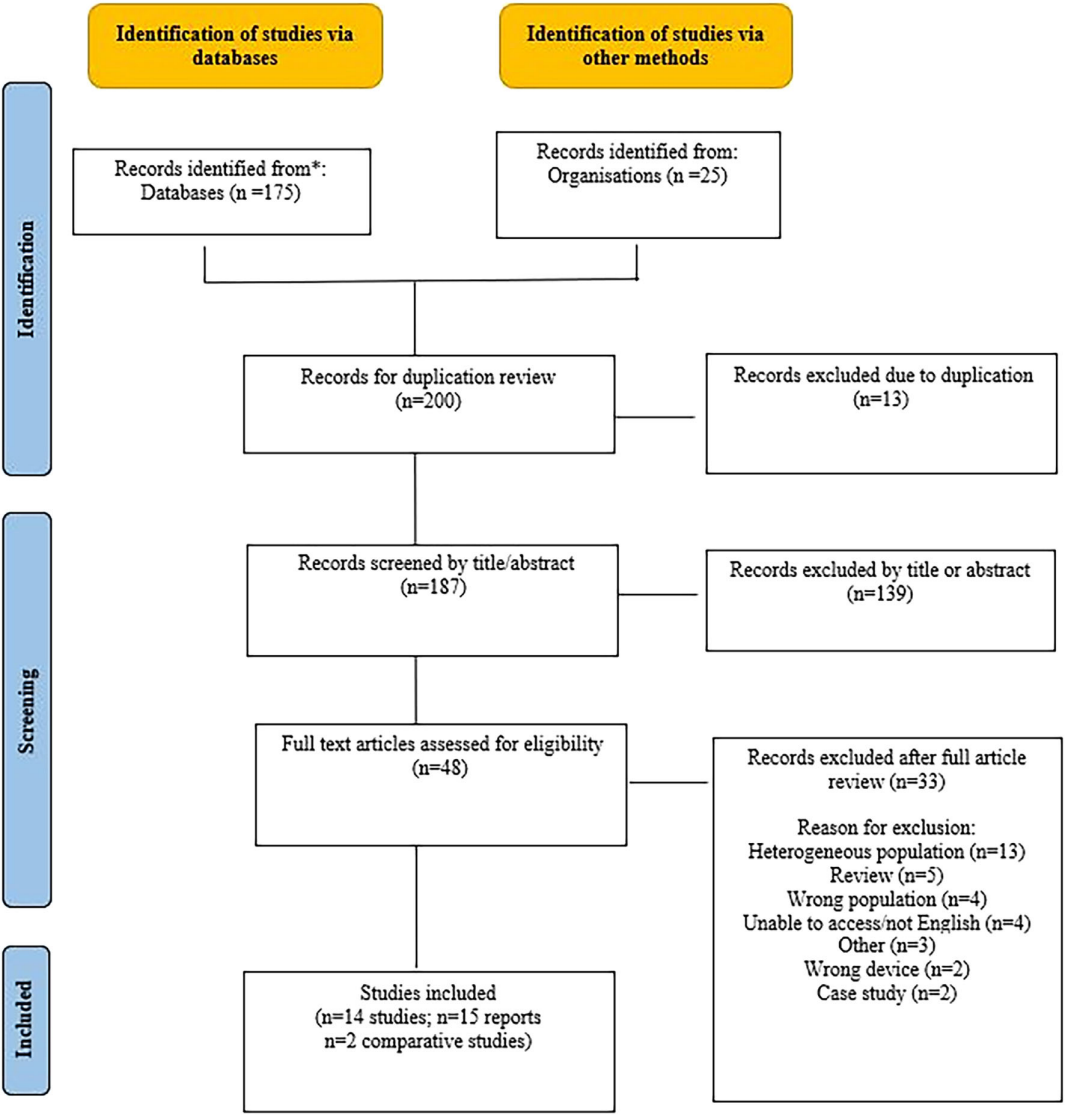
PRISMA diagram of systematic search.

A comprehensive literature search was conducted in MEDLINE (in June 2022), in line with the guidance of Cochrane and reported in line with the PRISMA.^9^ Search strings used are reported in Appendix S1 and the inclusion and exclusion criteria are presented in Table 1. The screening (title/abstract and full review) is reported in PRISMA (Figure 2). All identified studies (comparative and single arm) were extracted in standardised data tables.

**TABLE 1:** PICO(D) inclusion/exclusion criteria for systematic screening of identified studies.

PICO(D)	Inclusion criteria	Exclusion criteria
Population	People with extrinsic malignant ureteric obstruction	Benign extrinsic ureteric obstruction Intrinsic ureteric obstruction of any cause
Intervention	Resonance Metallic stent	Other metallic stents:
Comparator	Polyurethane JJ stents	Other types of interventions (nephrostomy, or surgical repair of the ureter).
Outcome	Primary patency Technical success Time to stent failure Stent failure rate Conversion to nephrostomy Infection incidence Other adverse events use Surgery time Mortality Quality of life Procedural protocols and follow‐up	
Study design	RCTs Prospective and retrospective comparator studies Single arm studies for Resonance.	Case studies Case series with <10 Reviews Opinion pieces Letters to editors
Status	Published papers only. Peer‐review journal articles only.	Non‐published literature Conference proceedings (conference, congress, symposium, or other meetings) including: ○ Posters ○ Abstracts from oral presentations
Date	All dates	No date restrictions

Abbreviation: PICO(D): Population, Intervention, Comparator, Design.

The literature review revealed a lack of high‐quality evidence in this area, no studies were randomised in design, and only two studies directly compared JJ stents with Resonance in patients with MUO, these were both based in Asia.^10^, ^11^ Quality assessment, based on the Newcastle‐Ottawa assessment scale (NOS) for assessing the quality of nonrandomised studies,^12^ revealed that the comparative study by Chow et al. contained higher quality data (Appendix S1) and the patient demographics were comparable with patients in other studies, including those in the United Kingdom. In addition, the study by Chow et al.^11^ limited confounding factors within the patient population, by reporting on the same patients, initially treated with an ordinary polyurethane JJ and then, following stent failure, treated with a Resonance metal stent. Finally, the monthly failure rate of the Resonance stent in the Chow et al.^11^ study (0.150) is higher than both pooled and individual results from other studies (pooled 0.025 per month; range 0.00–0.128; median 0.018; Appendix S1). Hence, in the absence of higher quality comparative studies between JJ and Resonance in the United Kingdom, Chow et al.^11^ are deemed suitable as a conservative estimate to inform the base case of the model.

### 2.3 Clinical Inputs

Clinical parameters and sources utilised are reported in Table 2 and were assumed to be a constant monthly hazard rate. The stent failure parameters were derived, using the functional duration of each stent from Chow et al.^11^ As the core study by Chow et al.^11^ ran beyond planned stent changes for both JJ (6 months) and Resonance (12 months), the data did not require extrapolation beyond the study duration. Hence, published Kaplan–Meier curves were digitised to estimate the mean monthly probability of failure for Resonance and JJ stents.^13^ At 12‐month intervals, the probability of Resonance still being functional was applied to account for planned stent changes of previous unchanged stents. The same process was applied at six monthly intervals for JJ stents.

**TABLE 2:** Base case, clinical parameters for Resonance and JJ.

Transition probabilities						
Parameter		Base value	Lower value	Upper value	Distribution	Source
All‐cause mortality		0.0505	0.0404	0.0606	Beta	± 20%, Appendix S3
Resonance	Stent failure (all states)	0.1502	0.1202	0.1802	Beta	± 20%,^11^
	Patent/failed to UTI	0.0205	0.0018	0.0547	Beta	± SD, Appendix S3
JJ	Stent failure (all states)	0.3648	0.2918	0.4378	Beta	± 20%,^11^
	Patent/failed to UTI	0.0085	0.0068	0.0102	Beta	± SD, Appendix S3

Utilities						
Parameter		Base value	Lower value	Upper value	Distribution	Source
Patent stent utility and planned change		0.76	0.684	0.836	Beta	± 10%,^14^
Patent stent + UTI utility		0.73	0.657	0.803	Beta	± 10%,^14^
Stent Failure		0.62	0.558	0.682	Beta	± 10%,^14^

Health resource use costs						
Parameter		Base case cost	Lower cost	Upper cost	Distribution	Source
Resonance	Insertion	£1565.12	£1316.73	£2137.19	Gamma	Appendix S4,^8^, ^15^
	UTI	£44.01	£28.48	£62.86	Gamma	± 20%,^8^
	Replacement planned	£1755.02	£1496.13	£2328.76	Gamma	Appendix S4,^8^, ^15^
	Replacement unplanned	£1931.02	£1672.13	£2769.76	Gamma	Appendix S3,^8^, ^15^
JJ	Insertion	£871.20	£689.14	£1393.26	Gamma	The literature^8^, ^15^
	UTI costs	£44.01	£28.48	£62.86	Gamma	± 20%,^8^
	Replacement planned	£1061.10	£868.54	£1584.83	Gamma	Appendix S4,^8^, ^15^
	Replacement unplanned	£1237.10	£1044.54	£2025.83	Gamma	Appendix S4,^8^, ^15^
Other						
Discount rate: costs		3.5%	‐	‐	Fixed	The literature^16^
Discount rate: outcomes		3.5%	‐	‐	Fixed	The literature^16^

The monthly probability of UTI for Resonance was calculated from the incidence of UTI and the total patient months follow‐up, pooled from multiple sources.^10^, ^16^, ^17^, ^18^ In line with previous economic analyses by NICE, the infection rate with JJ was assumed to be the same as with Resonance.^8^ Death was modelled based on the total pooled incidence of death in patient months,^10^, ^16^, ^17^, ^18^, ^19^, ^20^, ^21^, ^22^ used in addition to life UK life tables, weighted according to gender, and averaged over 5 years from age 57–62. Detailed calculation of transition probabilities can be found in Appendix S3.

The primary effectiveness measure was QALYs, which incorporates health‐related quality‐of‐life (HRQoL) and mortality. Utilities were estimated from a UK‐based study.^14^ The utility value for a patent stent was derived from the mean utility for the ‘stent group’, the lower 95% CI from the ‘lower urinary tract group’ informed utility for UTI utility and the lower 95% CI utility from a ‘stent group’ informed stent failure utility.^14^ All utilities were adapted for monthly cycles and applied to both arms of the model (Table 2).

### 2.4 Costs

Healthcare resources were estimated from bottom‐up costing as described in Table 2 and Appendix S2. The Personal Social Services Research Unit (PSSRU) 2021^23^ informed the costs for healthcare professionals and bed stay. Costs for CT scan, renogram and X‐ray were provided by the 2022/2023 tariff price. Theatre running costs were derived from Scottish public health financing files of 2021 and inflated to 2022/2023 prices.^24^ Consumable use and costs, along with JJ costs, were derived from previous models^15^ and inflated to 2022/2023 level prices using National Health Services Cost Inflation Index (NHSCII) prices.^25^ Resonance prices were provided by the company. The insertion procedure times of 21 and 20.6 min for Resonance and JJ, respectively, were derived from the literature.^26^ Due to a lack of literature on replacement time, based on these authors' clinical experience an addition of 10 min was applied to insertion time to estimate replacement time. In alignment with other models, recovery time was assumed for day case procedures, with a bed stay of 4 h and the inclusion of recovery nurse time comprising of 30 min one‐to‐one nurse time with a band 6 nurse and 240 min of a band 5 nurse shared with three other patients.^15^ Costs associated with UTI were assumed to be a visit to a general practitioner (GP)^23^ and a prescription for antibiotics. Finally, it was assumed that stent failure was associated with an unplanned visit to oncology outpatients with reference costs for palliative outpatient care with a specialist applied.

### 2.5 Cost‐effectiveness analysis

The results of the cost‐utility analysis are reported as incremental costs, incremental QALYs, incremental cost‐effectiveness ratio and net monetary benefit. Incremental costs and QALYs are the difference in costs and QALYS for the Resonance arm compared with the JJ arm. A negative incremental cost indicates a cost saving. Incremental cost‐effectiveness ratio (ICER) is the incremental difference in costs divided by the difference in QALYs. The ICER is reported against NICE's recommended threshold willingness to pay threshold (WTP) of £20 000 per QALY. A cost‐effectiveness acceptability curve is used to demonstrate uncertainty around cost‐effectiveness at varying WTP thresholds. Net monetary benefit (NMB) is calculated at specific WTP thresholds by multiplying the incremental difference in QALY by the WTP threshold and subtracting the incremental difference in costs. A positive NMB indicates cost‐effectiveness.

### 2.6 Sensitivity analysis

To assess the robustness of the model, deterministic and probabilistic sensitivity analyses were performed. Deterministic univariate sensitivity analysis was conducted by varying input parameters within plausible bounds, and the impact on the total cost differences and NMB are presented as tornado plots. Probabilistic sensitivity analysis (PSA), using a Monte Carlo simulation, was conducted to assess the simultaneous impact of uncertainty around key parameters. All cost and probability variables were included as well as mortality rate and utility values. Base case utilities and transition probabilities were varied by 20%, except for death which was varied by 10%, and UTI resolution also varied by 10% to prevent the upper bound from exceeding 1 (Table 2). Resource costs were varied by increasing and decreasing the procedure time for stent insertion and replacement (Appendix S4). In addition, the technology costs are varied using the lowest and highest available cost of Resonance on NHSSC and upper bands of polyurethane JJ are as reported previously, inflated to 2022/23 prices.^15^ In the absence of documented recovery information, and based on clinical feedback, the recovery time upper limit is set at 75% of the patients being day cases and 25% resulting in an overnight stay for both arms of the study, with staffing costs and bed stay cost impacted accordingly. For PSA, gamma distribution is applied to all costs, to reflect their right‐skewing nature; and beta distribution is used for utilities and transition probabilities, due to their bound between 0 and 1.^17^ The PSA was run for 1000 iterations, and incremental costs in Great British Pounds were plotted against incremental QALYs. The probability for Resonance to be cost‐effective is plotted on a cost‐effectiveness acceptability curve.

### 2.7 Scenario analysis

In scenario analysis, the effect of using different estimates for insertion times (37.5 min for Resonance and 22.5 min for JJ) and replacement times (67.5 min for Resonance and 52.5 min for JJ) was explored in line with previously published models in this therapy area^15^ (detailed in Appendices S5 and S6).

## 3 RESULTS

### 3.1 Cost‐effectiveness

Costs for using Resonance for the treatment of MUO were lower than the standard JJ at 1, 2, 3 and 5 years (Table 3). Resonance interventions incur −£726.53 less cost than JJ after 1 year (15.2% reduction) and −£2164.74 less cost than JJ after 5 years (23.4% reduction). Savings were driven by the reductions in repeat surgical interventions with Resonance use estimated to be associated with −1.88 fewer surgical interventions than JJ in the first year and −4.02 fewer over 5 years. The predominant costs, in both cohorts are on account of the management of stent replacements due to stent failure. Whereas planned stent changes account for a small proportion of the costs to the healthcare system.

**TABLE 3:** Base case costs, QALYS and stent exchanges over 1, 2, 3 and 5 years.

		Total costs	
	Resonance	JJ	Increment difference
Cost (year 1)	£4067.96	£4794.49	−£726.53
Cost (year 2)	£5627.20	£7099.96	−£1472.76
Cost (year 3)	£6435.97	£8295.80	−£1859.83
Cost (year 5)	£7073.07	£9237.81	−£2164.74
		Total QALYs	
	Resonance	JJ	Increment difference
QALYs (year 1)	0.565	0.543	+0.022
QALYs (year 2)	0.834	0.700	+0.034
QALYs (year 3)	0.981	0.941	+0.041
QALYs (year 5)	1.109	1.104	+0.046
		Total stents used	
	Resonance	JJ	Increment difference
No. of stent exchanges (year 1)	2.292	4.170	−1.877
No. of stent exchanges (year 2)	3.139	6.100	−2.962
No. of stent exchanges (year 3)	3.593	7.137	−3.544
No. of stent exchanges (year 5)	3.967	7.99	−4.024

The base case analysis indicates a gain of quality‐of‐life when using the metallic stent compared with JJ across the five‐year time horizon (Table 3) resulting in a QALY gain of 0.022 after 1 year and 0.046 QALYs after 5 years. With Resonance use reporting both a cost‐saving and a QALY gain compared with JJ stent use, the estimated Incremental Cost‐effectiveness Ratio (ICER) predicts Resonance as dominant over JJ stents and hence cost‐effective. Based on these costs and consequences, a net monetary benefit (NMB) of £3077.83 is estimated at a maximum willingness to pay threshold of £20 k per QALY.

**FIGURE 3:**
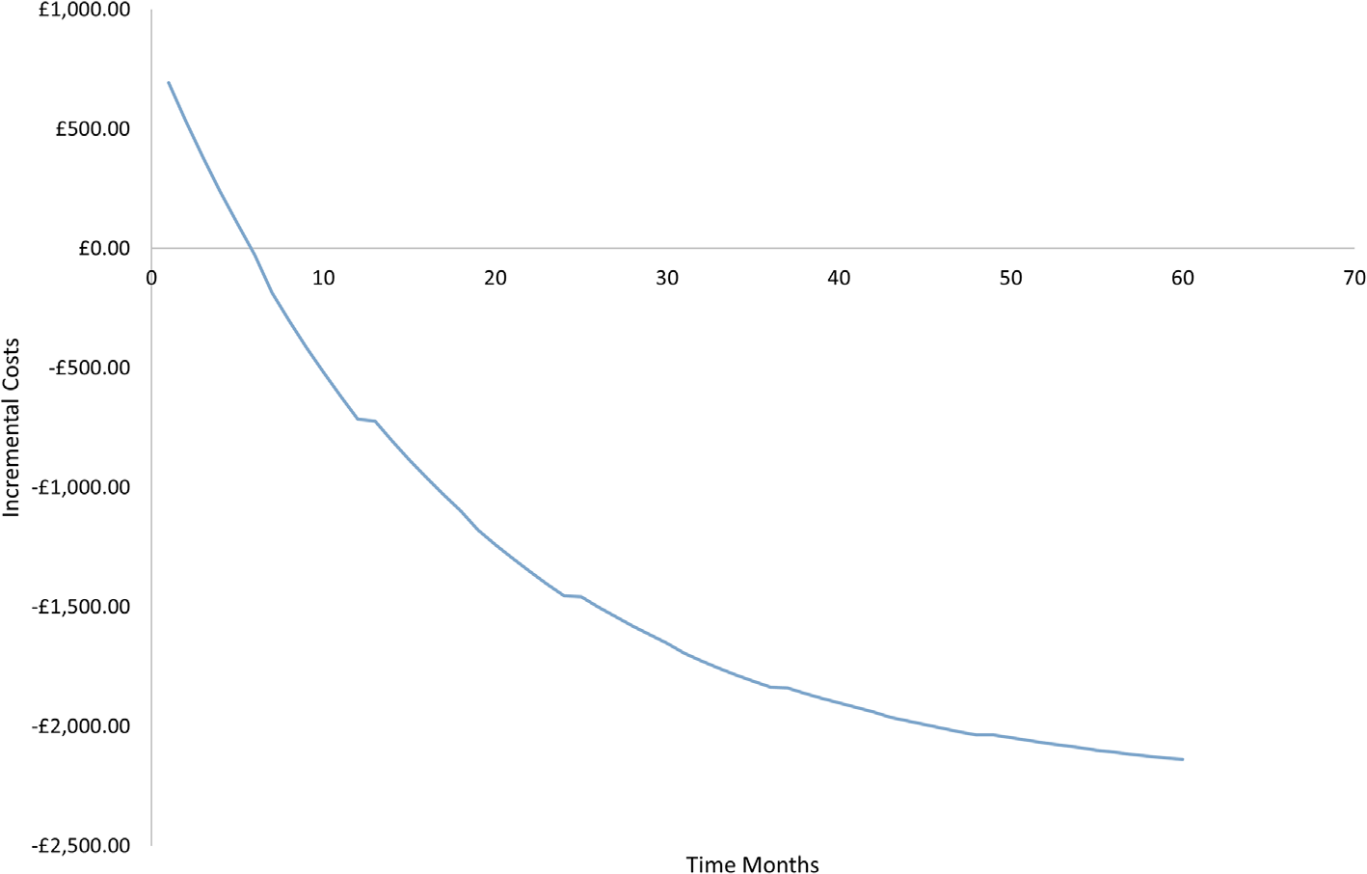
Incremental costs per patient for treatment with Resonance compared with JJ over a 5‐year. Time horizon. *y*‐axis reports the incremental differences in cost between Resonance use and JJ use (negative indicates a cost saving); *x*‐axis is time in months.

The impact over time was explored to account for initial increased insertion costs with Resonance, and the metal stent becomes cost‐neutral between 5 and 6 months and cost‐saving beyond 6 months (Figure 3).

### 3.2 Sensitivity analysis

**FIGURE 4:**
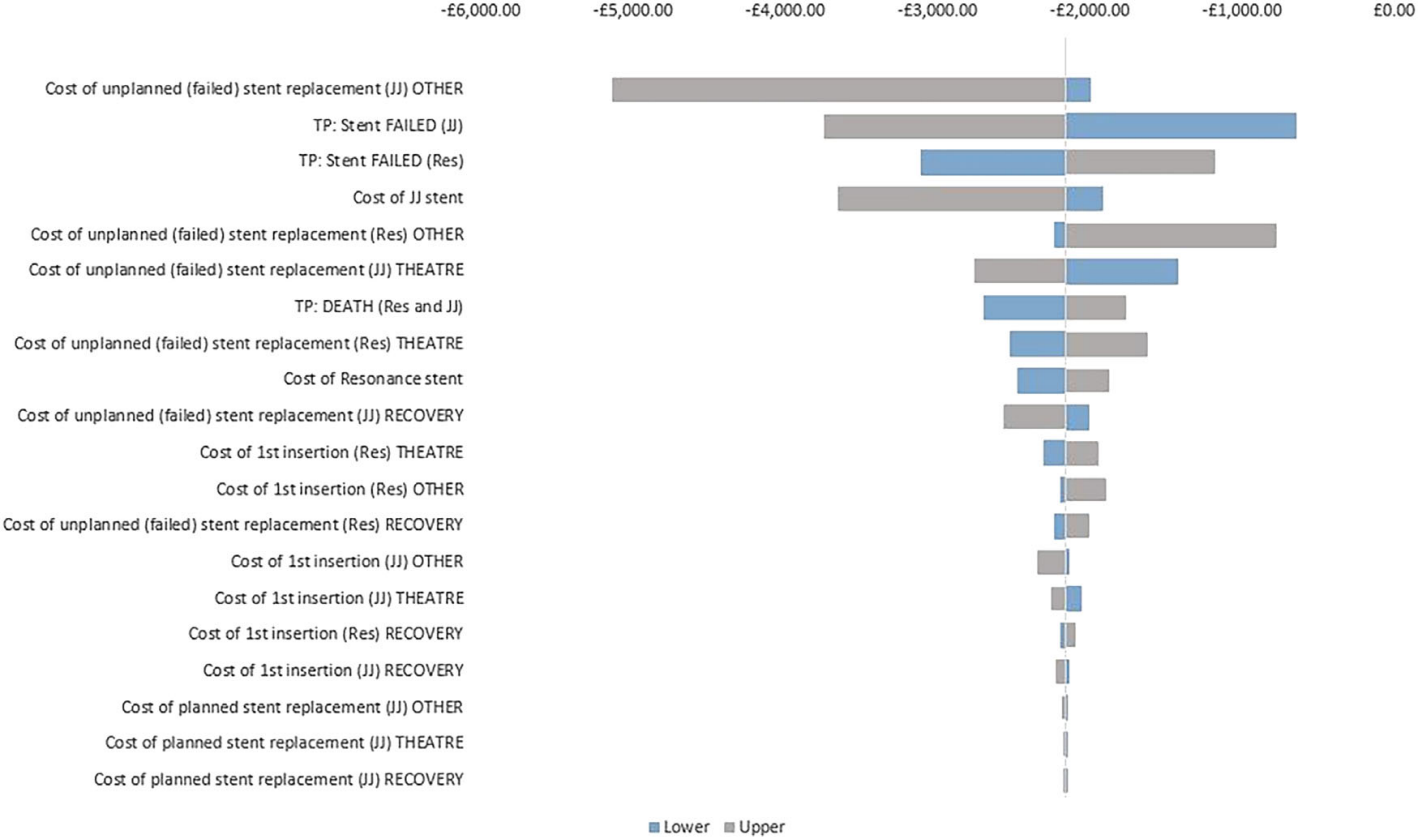
Tornado showing the influence of increasing or decreasing the top 15 key variables on Incremental costs. *y*‐axis lists the variables in order from highest impact to lowest impact on cost; *x*‐axis reports changes in incremental cost from the base case as each variable is varied to the lower and upper limit.

**FIGURE 5:**
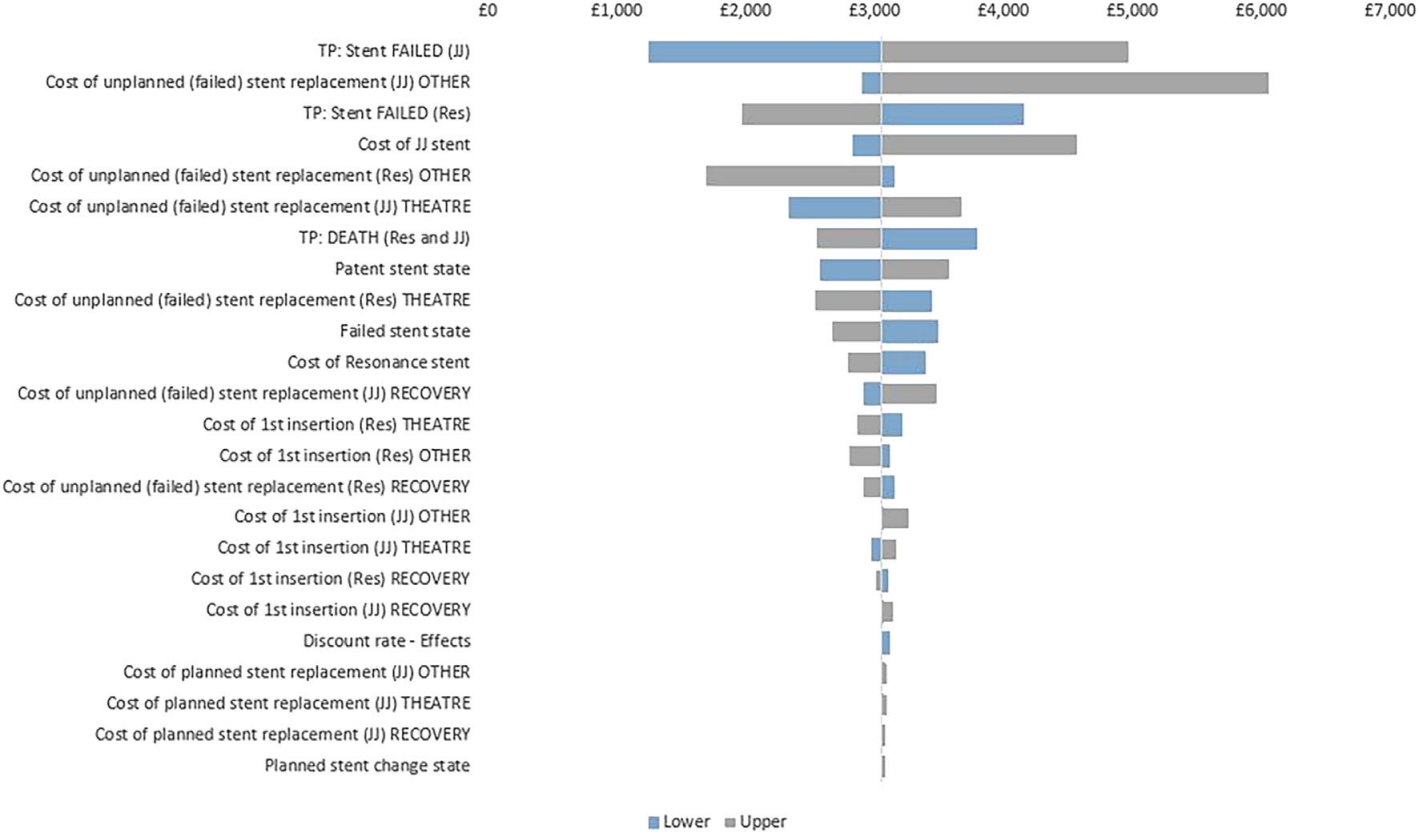
Tornado shows the influence of increasing or decreasing the top 15 key variables on net monetary benefit (NMB) at a willingness to pay threshold of £20 000 per QALY (quality adjusted life year). *y*‐axis lists the variables in order from highest impact to lowest impact on cost; *x*‐axis reports changes in incremental net monetary benefit from the base case as each variable is varied to the lower and upper limit.

The results for the deterministic sensitivity analysis are presented in tornado diagrams in Figures 4 and 5, where the central line indicates base case incremental costs and NMB, respectively. All parameter variations continued to return an incremental cost saving and a positive NMB for Resonance. The parameters exerting the most influence are the probabilities of JJ and Resonance stent failure, unplanned stent change costs, and JJ stent costs.

**FIGURE 6:**
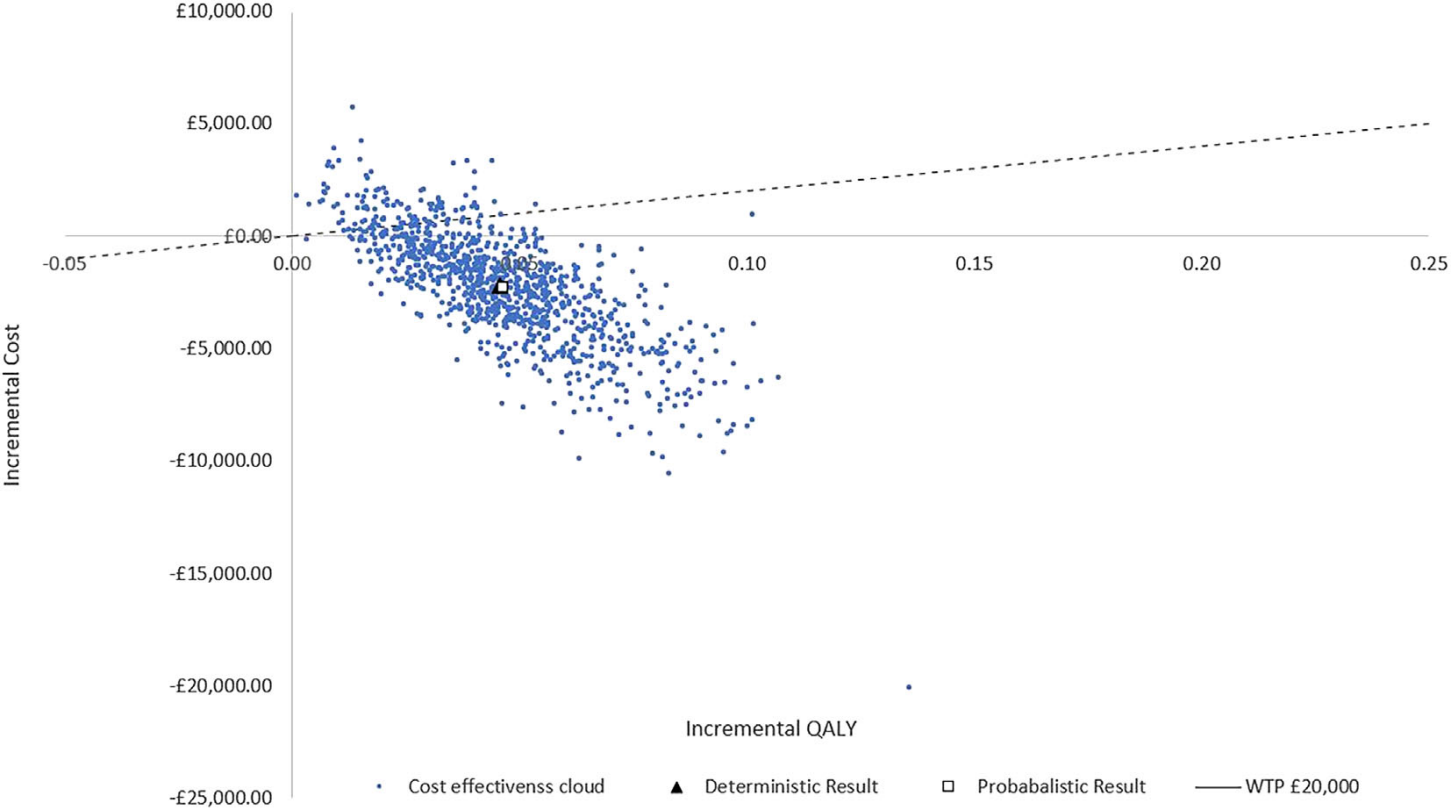
Cost‐effectiveness plane, demonstrating 1000 Monte Carlo simulations (dots) deterministic result (square) the probabilistic results (triangle) and willingness to pay threshold (dashed line).

**FIGURE 7:**
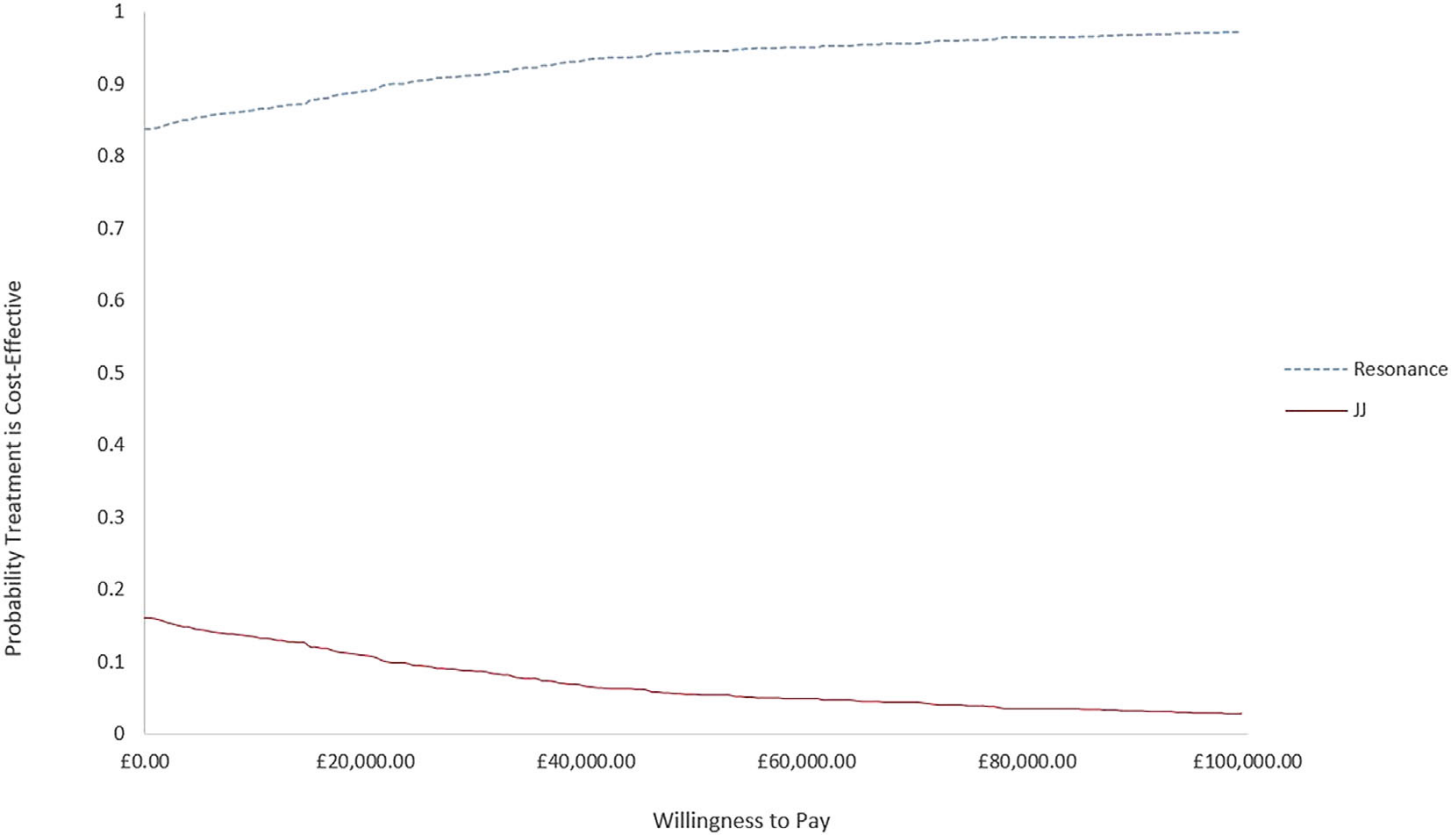
Cost‐effectiveness acceptability curve for Resonance and JJ stents for the treatment of MUO.

The results of 1000 Monte Carlo iterations are presented in Figure 6. At a willingness to pay (WTP) threshold of £20 000 per QALY, approximately 89.3% of the simulations are within this threshold. The average probabilistic estimate also predicts a dominant ICER in the southeast quadrant of the cost‐effectiveness plane. The cost‐effectiveness acceptability curve illustrates at a WTP of £0 Resonance has an 83.9% probability of being cost‐effective (Figure 7).

### 3.3 Scenario analysis

Scenario analysis, adopting different insertion and replacement times used in other models, continues to predict the same QALY gain as the base case and number of stent changes (as expected), whereas the incremental costs estimate an additional £1073.36 cost‐saving over the base case at 5 years. In this scenario, Resonance continues to be dominant over JJ stents (Table 4). NMB is predicted to increase over the base case and PSA analysis for this model estimates an 87.0% probability of Resonance being cost‐effective. Granular details of scenario analysis can be found in Appendix S5.

**TABLE 4:** Scenario analysis costs over 1, 2, 3 and 5.

		Total costs	
	Resonance	JJ	Increment difference
Cost (year 1)	£5831.67	£6907.65	−£1075.98
Cost (year 2)	£8130.64	£10 328.46	−£2197.82
Cost (year 3)	£9323.11	£12 102.82	−£2779.72
Cost (year 5)	£10 262.47	£13 500.57	−£3238.10

## 4 DISCUSSION

A potential limitation to the uptake of metallic stents, particularly for first‐line use, is the increased purchase cost of the device compared with polyurethane JJ stents. However, while the economic impact of using metallic stents over polyurethane JJ stents has been partially explored, there is a lack of robust economic evidence, particularly for the MUO cohort.

Several studies have attempted to uncover the economic impact of treating ureteric obstruction with Resonance or standard polyurethane JJ. However, these studies have focussed on benign patients alone, or have utilised a heterogeneous patient cohort comprised of benign and malignant diseases.^27^, ^28^, ^29^, ^30^ The economic analysis by Taylor^29^ is the only study to utilise individualised insertion costs for both JJ and Resonance, reporting median insertion costs of USD6072.75 and USD9469.50, respectively. However, data is then extrapolated with the assumption that JJ stents need exchanging every 3–4 months, whereas Resonance requires stent changing only at 12 months, no between‐planned stent changes are included. The authors give an annual procedure cost of USD18 000–36 000 for JJ and USD9469.50 for Resonance.^29^ Other economic analyses report the mean insertion costs, with the only difference between arms comprising the cost of the stent, these studies did not incorporate any other potential variances in the cost models.^27^, ^28^, ^30^ Of these analyses, Lopez‐Huertas^28^ included individual patient data for stent changes and maintenance costs for both polyurethane JJ and Resonance in their analysis over 12 months, reporting a 43% reduction in costs per patient‐year with the use of the metal stent.^28^ Similarly, Baumgarten^30^ reports savings of up to 59.5% per patient‐year, and Polcari 2010 presents a saving of 43% per patient year.^27^, ^30^ While all these analyses indicate that the Resonance stent is cost‐saving per patient‐year, in the current literature there is a lack of thorough economic analyses including assessment of long‐term impact, robustness, and inclusion of sensitivity analyses, particularly in the cohort of MUO. Hence, this economic analysis sought to answer a more focussed question in that it aims to evaluate the economic impact of one type of metal stent, (Resonance) which has broadly similar insertion and exchange techniques compared with standard polyurethane JJ stents, care in the UK healthcare system for MUO, explored from the National Health Service (NHS) provider's perspective.

The findings of this cost‐effectiveness analysis indicate that treatment of MUO with Resonance reduces the number of unplanned surgical interventions, compared to JJ stents, and this intervention is cost‐effective in the United Kingdom (ICER dominant). The 23.3% reduction in total costs predicted here is more conservative than the 43% and 59.5% reported previously in the United States (US).^28^, ^30^ While previous studies cannot be directly compared to this analysis due to differences in target populations, healthcare costs, and methodology, the report of Resonance being cost‐effective is consistent. The conservative nature of this current work is likely related to the inclusion of planned changes and the expectation of early stent changes due to failure. Other studies have looked at pooled data for metal stents and utilised assumptions around planned stent changes at 6 months with no risk for stent failure for JJ stents^8^ However, the literature indicates that polyurethane stents are changed more frequently than at 6‐month intervals and are prone to failure.^8^, ^28^ Thus, the lack of stent failures in the control arm puts other models at risk of underestimating the costs and overestimating QALYs of treatment with JJ stents and such methodology is at risk of underestimating any potential cost‐savings with metal stents and increases the uncertainty in the estimate of calculated ICER.

Deterministic and probabilistic sensitivity analyses confirmed the robustness of the initial findings. Unsurprisingly, cost‐effectiveness is most responsive to the probability of stent failure, which highlights the need for up‐to‐date established failure rate data. The DSA demonstrates that the incremental costs and NMB are largely unaffected by changes in the unit cost of the Resonance stent. Although the univariate DSA demonstrates that cost‐effectiveness is sensitive to several input parameters, at no point does the NMB become negative, nor does Resonance become cost‐incurring and the ICER remains dominant throughout. The PSA indicates that the probability of cost‐effectiveness is 89.3% at the UK WTP threshold of £20 000 per QALY gain, further, the ICER becomes dominant after 5 months of care. Scenario analysis revealed interesting results. Applying the insertion and replacement times and length of stay assumptions from previous work by NICE^8^ to the model continues to estimate a dominant ICER, with estimates of cost‐savings being higher than the base case. Further supporting the robustness and cost‐effectiveness nature of the Resonance metal stent over the JJ.

The current model is not without its limitations. Firstly, high‐quality evidence is lacking in this area; no studies were randomised and only two studies directly compared JJ stents with Resonance in patients with MUO.^10^, ^11^ Secondly, the core study population is Taiwanese, and while of a similar age and disease background, may differ in unobserved ways from a UK population. Thirdly, Chow et al.^11^ is retrospective with no strict follow‐up protocol, hence factors influencing stent duration may have been omitted. In addition, the use of the same patient cohort, while overcoming confounding with regards to patient demographics, introduces time‐varying issues due to the progressive nature of the malignant ureteral obstruction, thus the benefit of metallic stents might be underestimated. Fourthly this model assumes that stent failure results in the placement of the same type of stent, which may not be clinically relevant. Finally, the quality‐of‐life impact of this work is likely underestimated due to the lack of specific utility data in this patient cohort. The data are derived from patients without a malignant obstruction and hence may overestimate the disutility of a stent, as otherwise ‘healthy’ persons likely perceive any discomfort of a stent differently to MUO patients who require a stent to ensure chemotherapeutics can be administered.^1^, ^3^ In addition, any negative impact on utility following repeated general anaesthetic in a frail patient cohort has been unable to be quantified and hence not included in this model.

An obvious option to overcome these limitations would be a prospective randomised study, however, due to heterogeneity of the underlying disease and low patient numbers, such a study design would not be easily feasible. Indeed, given the limited evidence available, NICE has recommended routine collection of data on all ureteric stent placement procedures to perform real‐world evidence analysis,^15^ although no such national registry is currently available in the United Kingdom.

## 5 CONCLUSIONS

The fallout from the COVID‐19 pandemic has left a large elective backlog and a sizable efficiency target for the NHS over the next few years. Establishing efficient and effective treatment options for MUO is an opportunity to reduce surgical procedures for this frail population and the overstretched surgical services in the NHS. This work has demonstrated that the Resonance metallic stent could be a cost‐effective option for patients with MUO expected to survive beyond 6 months, by removing unnecessary, unplanned surgical re‐intervention, freeing capacity for elective provision and reducing readmission due to stent failure. However, uncertainties in data would benefit from national reporting of all ureteric stents enabling a clearer understanding of the economic impact of such devices specific to the UK market.

## AUTHOR CONTRIBUTIONS

**Dawn M. Cooper**: Conceptualisation (lead); formal analysis (lead); Data curation (lead); methodology (lead); writing—original raft (lead). **Rachel Lines**: Conceptualisation (support); validation (equal); writing—review and editing (equal). **Iqbal Shergill**: Conceptualisation (support); methodology (support); validation (equal); writing—review and editing (equal).

## CONFLICT OF INTEREST STATEMENT

DC is a salaried employee of Cook Medical, a Cook Group Company. RL and IS have no conflicts of interest.

## Supporting information


**Appendix S1.** Literature Search, NOS Assessment and Resonance data pooling.
**Appendix S2.** Detailed cost breakdown.
**Appendix S3.** Calculating Mean Probabilities.
**Appendix S4.** Data for Sensitivity Analysis.
**Appendix S5.** Scenario Analysis Data.

**Data S1.** Supporting Information.

## Data Availability

Additional data are not publicly available.

## References

[1] Khoo CC , Abboudi H , Cartwright R , El‐Husseiny T , Dasgupta R . Metallic ureteric stents in malignant ureteric obstruction: a systematic review. Urology. 2018;118:12–20. 10.1016/j.urology.2018.01.019 29408390

[2] Tabib C , Nethala D , Kozel Z , Okeke Z . Management and treatment options when facing malignant ureteral obstruction. Int J Urol. 2020;27(7):591–598. 10.1111/iju.14235 32253785

[3] Fiuk J , Bao Y , Calleary JG , Schwartz BF , Denstedt JD . The use of internal stents in chronic ureteral obstruction. J Urol. 2015;193(4):1092–1100. 10.1016/j.juro.2014.10.123 25463984

[4] Sountoulides P , Kaplan A , Kaufmann OG , Sofikitis N . Current status of metal stents for managing malignant ureteric obstruction. BJU Int. 2010;105(8):1066–1072. 10.1111/j.1464-410X.2009.09140.x 20067458

[5] Cordeiro MD , Coelho RF , Chade DC , Pessoa RR , Chaib MS , Colombo‐Júnior JR , et al. A prognostic model for survival after palliative urinary diversion for malignant ureteric obstruction: a prospective study of 208 patients. BJU Int. 2016;117(2):266–271. 10.1111/bju.12963 25327474

[6] Ganatra AM , Loughlin KR . The Management of Malignant Ureteral Obstruction Treated with ureteral stents. J Urol. 2005;174(6):2125–2128. 10.1097/01.ju.0000181807.56114.b7 16280741

[7] Williams KG , Blacker AJ , Kumar P . Ureteric stents the past, present and future. J Clin Urol. 2018;11(4):280–284. 10.1177/2051415817722934

[8] Eaton Turner E , Jenks M , McCool R , Marshall C , Millar L , Wood H , et al. The Memokath‐051 stent for the treatment of ureteric obstruction: a NICE medical technology guidance. Appl Health Econ Health Policy. 2018;16(4):445–464. 10.1007/s40258-018-0389-3 29616460 PMC6028873

[9] Page MJ , McKenzie JE , Bossuyt PM , Boutron I , Hoffmann TC , Mulrow CD , et al. The PRISMA 2020 statement: an updated guideline for reporting systematic reviews. BMJ. 2021;29:n71. 10.1136/bmj.n71 PMC8005924 33782057

[10] Chen Y , Liu CY , Zhang ZH , Xu PC , Chen DG , Fan XH , et al. Malignant ureteral obstruction: experience and comparative analysis of metallic versus ordinary polymer ureteral stents. World J Surg Oncol. 2019;17(1):74, 74. 10.1186/s12957-019-1608-6 31039812 PMC6492337

[11] Chow PM , Chiang IN , Chen CY , Huang KH , Hsu JS , Wang SM , et al. Malignant ureteral obstruction: functional duration of metallic versus polymeric ureteral stents. PLoS ONE. 2015;10(8):e0135566. 10.1371/journal.pone.0135566 26267140 PMC4534199

[12] Wells GA , Shea B , O'Connell D , Peterson J , Welch V , Losos M , et al. The Newcastle‐Ottawa scale (NOS) for assessing the quality of nonrandomised studies in meta‐analysis. 2021.

[13] Bounthavong M. Incremental thoughts the choice Institute student blog. 2018 [cited 2022 Dec 2]. Generating Survival Curves from Study Data: An Application for Markov Model. Available from: https://choiceblog.org/2018/03/23/generating-survival-curves-from-study-data-an-application-for-markov-models/

[14] Joshi HB , Stainthorpe A , MacDonagh RP , Keeley FX , Timoney AG . Indwelling ureteral stents: evaluation of symptoms, quality of life and utility. J Urol. 2003;169(3):1065–1069. 10.1097/01.ju.0000048980.33855.90 12576847

[15] NICE . Memokath‐051 stent for ureteric obstruction Medical technologies guidance [MTG35 [Internet]. 2018 [cited 2022 Dec 1]. Available from: https://www.nice.org.uk/guidance/mtg35/chapter/3-Evidence

[16] Kang Q , Jiang F , Yu Y , Shen C , Lv H , Yang B . Application of Resonance metallic stents for malignant ureteral obstruction. Minim Invasive Ther Allied Technol. 2018;27(6):333–338. 10.1080/13645706.2018.1443944 29475395

[17] Drummond MF , Sculpher MJ , Claxton K , Stoddart GL , Torrance GW . Methods for the economic evaluation of health care programmes New York: Oxford University Press; 2015.

[18] Miyazaki J , Onozawa M , Takahashi S , Maekawa Y , Yasuda M , Wada K , et al. The Resonance® metallic ureteral stent in the treatment of malignant ureteral obstruction: a prospective observational study. BMC Urol. 2019;19(1):137. 10.1186/s12894-019-0569-y 31881875 PMC6935232

[19] Ho BSH , Chiu PKF , Lam W , Wong JHF , Wong CKW , Lai TCT , et al. Risk factors in the prediction of long‐term patency of Resonance metallic ureteric stent in malignant ureteric obstruction. BJUI Compass. 2020;1(2):74–81. 10.1002/bco2.14 35474710 PMC8988516

[20] Abbasi A , Wyre HW , Ogan K . Use of full‐length metallic stents in malignant ureteral obstruction. J Endourol. 2013;27(5):640–645. 10.1089/end.2012.0448 23237309

[21] Asakawa J , Iguchi T , Tamada S , Ninomiya N , Kato M , Yamasaki T , et al. Outcomes of indwelling metallic stents for malignant extrinsic ureteral obstruction. Int J Urol. 2018;25(3):258–262. 10.1111/iju.13500 29194771

[22] Goldsmith ZG , Wang AJ , Bañez LL , Lipkin ME , Ferrandino MN , Preminger GM , et al. Outcomes of metallic stents for malignant ureteral obstruction. J Urol. 2012;188(3):851–855. 10.1016/j.juro.2012.04.113 22819410

[23] Jones K , Burns A . Unit Costs of Health and Social Care 2021 Canterbury; 2021.

[24] Public Health Scotland . Scottish Financial Reports 2019/2020 [Internet]. 2020 [cited 2022 Dec 1]. Available from: https://beta.isdscotland.org/topics/finance/file-listings-fy-2019-to-2020/

[25] NHS England . National Cost Collection for the NHS [Internet]. London; 2021 [cited 2022 Dec 1]. Available from: https://www.england.nhs.uk/costing-in-the-nhs/national-cost-collection/

[26] Patel C , Loughran D , Jones R , Abdulmajed M , Shergill I . The Resonance® metallic ureteric stent in the treatment of chronic ureteric obstruction: a safety and efficacy analysis from a contemporary clinical series. BMC Urol. 2017;17(1):16. 10.1186/s12894-017-0204-8 28283031 PMC5345181

[27] Polcari AJ , Hugen CM , López‐Huertas HL , Turk TM . Cost analysis and clinical applicability of the Resonance metallic ureteral stent. Expert Rev Pharmacoecon Outcomes Res. 2010;(1):11–15. 10.1586/erp.09.74 20121560

[28] López‐Huertas HL , Polcari AJ , Acosta‐Miranda A , Turk TMT . Metallic ureteral stents: a cost‐effective method of managing benign upper tract obstruction. J Endourol. 2010;24(3):483–485. 10.1089/end.2009.0192 20210650

[29] Taylor ER , Benson AD , Schwartz BF . Cost analysis of metallic ureteral stents with 12 months of follow‐up. J Endourol. 2012;26(7):917–921. 10.1089/end.2011.0481 22360415

[30] Baumgarten AS , Hakky TS , Carrion RE , Lockhart JL , Spiess PE . A single‐institution experience with metallic ureteral stents: a cost‐effective method of managing deficiencies in ureteral drainage. Int Braz J Urol. 2014;40(2):225–231. 10.1590/S1677-5538.IBJU.2014.02.13 24856490

