# Supplementary material for: Cost‐effectiveness of Resonance® metallic ureteral stent compared with standard polyurethane ureteral stents in malignant ureteric obstruction: A cost‐utility analysis
Source: BJUI Compass. 2024 Mar 20;5(5):465–75. doi: 10.1002/bco2.332 (PMC11090770; doi:10.1002/bco2.332)
Supplement: Supplementary file 1 — Appendix S1. Literature Search, NOS Assessment and Resonance data pooling. Appendix S2. Detailed cost breakdown. Appendix S3. Calculating Mean Probabilities. Appendix S4. Data for Sensitivity Analysis. Appendix S5. Scenario Analysis Data. [file BCO2-5-465-s001.docx]

# Appendix 1 Literature Search, NOS Assessment and Resonance data pooling

| Search Terms MEDLINE | | |
| --- | --- | --- |
| Search Number | Search Terms | Articles identified |
| #1 | Resonance and “cook medical” | 17 |
| #2 | “Resonance ureteral stent” | 4 |
| #3 | “Resonance stent” | 30 |
| #4 | metal AND ureteral AND stent | 184 |
| #5 | “Metal ureteral stent” | 14 |
| #6 | (#1 OR #2 OR #3 OR #4 OR #5) | 207 |
| #7 | Exp animals/ not humans | 4,752,531 |
| #8 | #6 NOT #7 | 189 |
| #9 | “Magnetic resonance” | 856,915 |
| #10 | #8 NOT #9 | 175 |

| **Newcastle-Ottawa Scale for Assessment of Comparative Studies** | | | |
| --- | --- | --- | --- |
|  | Selection  (Max 4 stars) | Comparability  (Max 2 stars) | Outcome  (Max 3 stars) |
| Chow et al 2015 | *** | ** | *** |
| Chen et al 2019 | *** | - | ** |

# Resonance stent failure rate, data pooling

|  |  |  |  |  |  |  |
| --- | --- | --- | --- | --- | --- | --- |
| Study | **Mean follow-up (months)** | Number of patients | Estimated total number of patient months | Total stent changes due to “failure” | Monthly Rate of failure | Monthly probability of failure |
| Wah (2007 | 6.4 | 15 | 96 | 0 | 0 | 0 |
| Goldsmith (2012) | 3.5 | 25 | 87.5 | 12 | 0.1371 | 0.1282 |
| Abassi (2013) | 12.3 | 20 | 246 | 8 | 0.0325 | 0.0320 |
| Kang (2018) | 4.8 | 14 | 67.2 | 0 | 0 | 0 |
| Miyaskai (2019) | 11.7 | 50 | 585 | 8.6 | 0.0147 | 0.0146 |
| Khoo (2021) | 12.1 | 39 | 471.9 | 11 | 0.0233 | 0.0230 |
| **Pooled** | | | **1553.6** | **39.6** | **0.0255** | **0.0251** |

# Appendix 2 Detailed cost breakdown

| **Base Case Insertion Costs** | | | | | | |
| --- | --- | --- | --- | --- | --- | --- |
|  |  | Resonance | | JJ | | Sources |
|  | Hourly cost | Time required | Cost | Time required | Cost |  |
| Anaesthetist | £123.00 | 21 minutes | £43.05 | 20.6 minutes | £42.23 | (12,15) |
| Surgeon | £122.00 | 21 minutes | £42.70 | 20.6 minutes | £41.89 | (12,15) |
| Registrar | £52 | 21 minutes | £18.20 | 20.6 minutes | £17.85 | (12,15) |
| Band 7 scrub | £51.00 | 21 minutes | £21.70 | 20.6 minutes | £21.29 | (12,15) |
| Band 5 scrub | £41.00 | 21 minutes | £14.35 | 20.6 minutes | £14.08 | (12,15) |
| Band 5/6 ODP | £46.00^¥^ | 21 minutes | £16.11 | 20.6 minutes | £15.49 | (12,15) |
| Band 3 circulating | £29.50 | 21 minutes | £10.33 | 20.6 minutes | £10.33 | (12,15) |
| Band 2 porters | £26.50 | 21 minutes | £9.28 | 20.6 minutes | £9.28 | (12,15) |
| **TOTAL HCPs** |  |  | **£175.70** |  | **£172.35** |  |
| Theatre running cost | £637.00 | 21 minutes | £223.1 | 20.6 minutes | £218.85 | (26) |
| **TOTAL theatre cost** |  |  | **£398.80** |  | **£391.20** |  |
| Band 6 recovery | £51.00 | 20 minutes | £17.00 | 20 minutes | £17.00 | (27,28) |
| Band 5 recovery | £41.00 | 80 minutes | £54.67 | 80 minutes | £54.67 | (27,28) |
| Bed stay | £288.00 | 4 hours | £48.00 | 4 hours | £48.00 | (27,28) |
| **RECOVERY TOTAL** |  |  | **£119.67** |  | **£119.67** |  |
| Device and Insertion consumables |  |  | £802.47 |  | £115.06 | (27,28) |
| Diagnostic contrast fluoroscopy |  |  | £109.00 |  | £109.00 | (27,28) |
| Prophylactics antibiotics |  |  | £24.11 |  | £24.11 | (27,28) |
| Insertion X-ray |  |  | £31.30 |  | £31.30 | (27,28) |
| CT scan |  |  | £79.78 |  | £79.78 | (27,28) |
| **TOTAL OTHER** |  |  | **£1,046.66** |  | **£360.33** |  |
| **OVERALL TOTAL** |  |  | **£1,565.12** |  | **£871.20** |  |

*¥ average of band 5 (£41) and band 6 £51) anaesthetic assistant. NHS Supply Chain Catalogue*

| **Base Case Replacement Costs** | | | | | | |
| --- | --- | --- | --- | --- | --- | --- |
|  |  | Resonance | | JJ | | Sources |
|  | Hourly cost | Time required | Cost | Time required | Cost |  |
| Anaesthetist | £123.00 | 31 minutes | £63.55 | 30.6 minutes | £62.73 | (12,15) |
| Surgeon | £122.00 | 31 minutes | £63.03 | 30.6 minutes | £62.22 | (12,15) |
| Registrar | £52.00 | 31 minutes | £26.87 | 30.6 minutes | £26.52 | (12,15) |
| Band 7 scrub | £51.00 | 31 minutes | £32.03 | 30.6 minutes | £31.62 | (12,15) |
| Band 5 scrub | £41.00 | 31 minutes | £21.18 | 30.6 minutes | £20.91 | (12,15) |
| Band 5/6 ODP | £46.00^¥^ | 31 minutes | £23.77 | 30.6 minutes | £23.46 | (12,15) |
| Band 3 circulating | £29.50 | 31 minutes | £15.24 | 30.6 minutes | £15.05 | (12,15) |
| Band 2 porters | £26.50 | 31 minutes | £13.69 | 30.6 minutes | £13.52 | (12,15) |
| **TOTAL HCPs** |  |  | **£259.37** |  | **£256.02** |  |
| Theatre running cost | £637.00 | 31 minutes | £329.33 | 30.6 minutes | £325.08 | (26) |
| **TOTAL theatre cost** |  |  | **£588.70** |  | **£581.10** |  |
| Band 6 recovery | £51.00 | 20 minutes | £17.00 | 20 minutes | £17.00 | (27,28) |
| Band 5 recovery | £41.00 | 80 minutes | £54.67 | 80 minutes | £54.67 | (27,28) |
| Bed stay | £288.00 | 4 hours | £48.00 | 4 hours | £48.00 | (27,28) |
| **RECOVERY TOTAL** |  |  | **£119.67** |  | **£119.67** |  |
| Device and Insertion consumables |  |  | £802.47 |  | £115.06 | (27,28) |
| Diagnostic contrast fluoroscopy |  |  | £109 |  | £109.00 | (27,28) |
| Prophylactics antibiotics |  |  | £24.11 |  | £24.11 | (27,28) |
| Insertion X-ray |  |  | £31.30 |  | £31.30 | (27,28) |
| CT scan |  |  | £79.78 |  | £79.78 | (27,28) |
| **TOTAL OTHER** |  |  | **£1,046.66** |  | **£360.33** |  |
| Oncology visit (stent fail) @ outpatient cost |  |  | £176.00 |  | £176.00 | (25) |
| **Additional stent failure cost** |  |  | **£176.00** |  | **£176.00** |  |
| **OVERALL TOTAL (planned)** |  |  | **£1,755.02** |  | **£1,061.10** |  |
| **OVERALL TOTAL (unplanned)** |  |  | **£1,931.02** |  | **£1,237.10** |  |

*¥ average of band 5 (£41) and band 6 (£51) anaesthetic assistant,*

# Appendix 3 Calculating Mean Probabilities

| **Resonance Stent Failure** | | | |
| --- | --- | --- | --- |
| **Start time** | **Survival probability S(t)** | **Failure probability S(t)** | **Number at risk R(t)** |
| 0 | 1 | - | 42.0 |
| 1 | 0.83920444 | 0.16079556 | 35.2 |
| 2 | 0.70316789 | 0.16210181 | 29.5 |
| 3 | 0.61294371 | 0.12831101 | 25.7 |
| 4 | 0.59309557 | 0.03238166 | 24.9 |
| 5 | 0.54787862 | 0.0762389 | 23.0 |
| 6 | 0.44676972 | 0.18454616 | 18.8 |
| 7 | 0.37592043 | 0.15858124 | 15.8 |
| 8 | 0.37007568 | 0.01554784 | 15.5 |
| 9 | 0.3255904 | 0.12020591 | 13.7 |
| 10 | 0.24316575 | 0.2531544 | 10.2 |
| 11 | 0.19119355 | 0.21373159 | 8.0 |
| 12 | 0.13444719 | 0.29680058 | 5.6 |
| **Mean** | **-** | **0.15019972** | **-** |
| Median | - | 0.159688397 | - |

| **JJ Stent Failure** | | | | |
| --- | --- | --- | --- | --- |
| **Start time** | **Survival probability S(t)** | **Failure probability S(t)** | **Estimated S(t)**  **S(t) = exp(-**λ**t)** | **Number at risk R(t)** |
| 0 | 1 |  | 1.0000 | 42.000 |
| 1 | 0.74873835 | 0.25126165 | 0.6504 | 31.447 |
| 2 | 0.47419245 | 0.36667803 | 0.4230 | 19.916 |
| 3 | 0.31643415 | 0.33268834 | 0.2751 | 13.290 |
| 4 | 0.14708704 | 0.53517331 | 0.1789 | 6.178 |
| 5 | 0.11898 | 0.1910912 | 0.1164 | 4.997 |
| 6 | 0.05807 | 0.51193478 | 0.0757 | 2.439 |
| **Mean** | **-** | **0.364804551** | **-** | **-** |
| Median | - | 0.349683186 | - | - |

| Urinary Tract Infections | | | | | | |
| --- | --- | --- | --- | --- | --- | --- |
|  | (n) infection | (N) patients | Follow-up (months) | Estimated total patient months | Monthly Infection rate | Monthly Probability infection |
| Chen et al 2019 | 4 | 30 | 12 | 360 | 0.011111111 | **0.011049611** |
| Abassi et al 2013 | 1 | 20 | 9.23 | 184.6 | 0.005417118 | **0.005402472** |
| Chow 2014 et al | 24 | 74 | 5.77 | 426.98 | 0.056208722 | **0.054658198** |
| Kang et al 2018 | 3 | 14 | 4.8 | 67.2 | 0.044642857 | **0.04366103** |
| Miyazki et al 2019 | 1 | 46 | 12 | 552 | 0.001811594 | **0.001809954** |
| TOTAL | 33 | 184 |  | 1590.78 |  |  |
|  |  |  | Monthly rate | 0.02074454 | Min | 0.001809954 |
|  |  | Monthly probability | | **0.020530853** | Max | 0.054658198 |

**Calculation 1.1**

Rate = # of events that occurred in a time period = UTI incidence

Total time period experienced by all subjects followed Total patient months

**Calculation 1.2**

Probability = 1 – exp ^(-rt)^

*p=probability, t = time, r=rate*

# All-Cause Mortality

Multiple studies reported incidence of mortality in the patient cohort of MUO (table G1). This data, utilising calculation 1.1 and 1.2 were used to determine estimated monthly probability of mortality. In addition to monthly probability based on incidence of death, UK life tables [[National life tables – life expectancy in the UK - Office for National Statistics (ons.gov.uk)](https://www.ons.gov.uk/peoplepopulationandcommunity/birthsdeathsandmarriages/lifeexpectancies/bulletins/nationallifetablesunitedkingdom/2018to2020)] (Table G2)were used to incorporate mean age related risk of death for 57-62 year olds, weighted based on male:female ratio in the Chow et al 2015 study. Finally these two probabilities were added together to inform all cause of death transition probability.

| **All Cause Death MUO patients** | | | | | | | | | | | | |
| --- | --- | --- | --- | --- | --- | --- | --- | --- | --- | --- | --- | --- |
| Author year | | Incidence of death | | Number of patients | | Mean/median follow up /time to death (months) | | | | Total patient months | | |
| Chen (2019) | | 10 | | 76 | | 11.8 | | | | 896.9 | | |
| Ho (2020) | | 50 | | 95 | | 9.04 | | | | 858.8 | | |
| Abassi (2013) | | 16 | | 20 | | 9.23 | | | | 184.6 | | |
| Kang 2018 | | 4 | | 14 | | 4.8 | | | | 67.2 | | |
| Miyaskai (2019) | | 24 | | 46 | | 11.7 | | | | 538.2 | | |
| Asakawa (2017) | | 46 | | 52 | | 6.9 | | | | 358.8 | | |
| Goldsmith (2012) | | 3 | | 25 | | 3 | | | | 75 | | |
| Total incidence of death | | 153 | | Patient months | | | | | | 2979.4 | | |
|  | |  | | Monthly rate of death | | | | | | 0.051352621 | | |
|  | |  | | **Monthly probability of death** | | | | | | **0.050056359** | | |
|  | |  | |  | |  | | | |  | | |
|  | **National life tables** | | | | | | | | | | |  |
|  | age | | mx | | qx | | lx | | dx | | ex |  |
| Male | 57 | | 0.005899 | | 0.005882 | | 92786.2 | | 545.7 | | 25.24 |  |
|  | 58 | | 0.006545 | | 0.006523 | | 92240.4 | | 601.7 | | 24.38 |  |
|  | 59 | | 0.007059 | | 0.007035 | | 91638.7 | | 644.6 | | 23.54 |  |
|  | 60 | | 0.007727 | | 0.007698 | | 90994.1 | | 700.5 | | 22.70 |  |
|  | 61 | | 0.008389 | | 0.008354 | | 90293.6 | | 754.3 | | 21.87 |  |
|  | 62 | | 0.009371 | | 0.009328 | | 89539.3 | | 835.2 | | 21.05 |  |
|  | Cumulative (nqx, 57-62) | | - | | 0.035619 | | - | | - | | - |  |
|  | Weighted cumulative (14/42) | | - | | 0.011873 | | - | | - | | - |  |
|  | age | | mx | | qx | | | lx | dx | | ex |  |
| Female | 57 | | 0.003790 | | 0.003783 | | | 95445.5 | 361.0 | | 28.10 |  |
|  | 58 | | 0.004219 | | 0.004210 | | | 95084.4 | 400.3 | | 27.20 |  |
|  | 59 | | 0.004492 | | 0.004482 | | | 94684.2 | 424.3 | | 26.31 |  |
|  | 60 | | 0.005056 | | 0.005043 | | | 94259.8 | 475.3 | | 25.43 |  |
|  | 61 | | 0.005439 | | 0.005424 | | | 93784.5 | 508.7 | | 24.56 |  |
|  | 62 | | 0.006254 | | 0.006235 | | | 93275.8 | 581.5 | | 23.69 |  |
|  | Cumulative (nqx, 57-62 years) | | - | | 0.022942 | | | - | - | | - |  |
|  | Weighted cumulative (28/42) | | - | | 0.015294667 | | | - | - | | - |  |
|  | Sum of weighted nqx (male and female) | |  | | 0.27167667 | | |  |  | |  |  |
|  | **Monthly probability of death** | |  | | **0.000459059** | | |  |  | |  |  |

*qx = Proportion dying each year, the conditional probability that an individual dies between age x and x+1, lx = exact number of individuals alive at each age, dx = deaths, the number of individuals dying within each age interval, ex = the average number of additional years at age x, hx =hazard rate, nqx(x-y) = Cumulative qx for time period (x-y)*

**All cause death transition probability = 0.050056359 + 0.000459059 = 0.050515418**

# Appendix 4 Data for Sensitivity Analysis

|  | **Resonance Insertion Base Case** | | | | | | | | |
| --- | --- | --- | --- | --- | --- | --- | --- | --- | --- |
|  |  | **Base case** | | | **Lower** | | **Upper** | | **Sources** |
|  | **Hourly cost** | **Time required** | **Cost** | | **Time required** | **Cost** | **Time required** | **Cost** |  |
| Anaesthetist | £123.00 | 21 minutes | £43.05 | | 15.6 minutes | £31.98 | 31 minutes | £63.55 | (15) ^b^ |
| Surgeon | £122.00 | 21 minutes | £42.70 | | 15.6 minutes | £31.72 | 31 minutes | £63.03 | (15) ^b^ |
| Registrar | £52.00 | 21 minutes | £18.20 | | N/A | £0.00 | 31 minutes | £26.87 | (15) ^b^ |
| Band 7 Scrub | £62.00 | 21 minutes | £21.70 | | N/A | £0.00 | 31 minutes | £32.03 | (15) ^b^ |
| Band 6 scrub | £51.00 | N/A | £0.00 | | 15.6 minutes | £13.26 | 31 minutes | £26.35 | (15) ^b^ |
| Band 5 scrub | £41.00 | 21 minutes | £14.35 | | 15.6 minutes | £10.66 | N/A | £0.00 | (15) ^b^ |
| Band 6 ODP^a^ | £51.00 | 10.5 minutes | £8.93 | | 7.8 minutes | £6.63 | 31 minutes | £13.18 | (15) ^b^ |
| Band 5 ODP^a^ | £41.00 | 10.5 minutes | £7.18 | | 7.8 minutes | £5.33 | 31 minutes | £10.59 | (15) ^b^ |
| Band 3 circulating | £26.50 | 21 minutes | £10.33 | | 15.6 minutes | £7.67 | 31 minutes | £15.24 | (15) ^b^ |
| Band 2 porters | £26.50 | 21 minutes | £9.28 | | 15.6 minutes | £6.89 | 31 minutes | £13.69 | (15) ^b^ |
| **TOTAL HCPs** | |  | **£175.70** | |  | **£114.14** |  | **£264.53** |  |
| Theatre running cost | £637.00 | 21 minutes | £223.10 | | 15.6 minutes | £165.73 | 31 minutes | £329.33 | (26) |
| **TOTAL theatre cost** | |  | **£398.80** | |  | **£279.87** |  | **£593.86** |  |
| Band 6 recovery | £51.00 | 20 minutes | £17.00 | | 20 minutes | £17.00 | 20 minutes | £17.00 | (27,28) |
| Band 5 recovery | £41.00 | 80 minutes | £54.67 | | 80 minutes | £54.67 | 80 minutes | £54.67 | (27,28) |
| Bed stay | £288.00 | 4 hours | | £48.00 | 2 hours | £24.00 | 9 hours | £108.00 | (27,28) |
| **RECOVERY TOTAL** | |  | | **£119.67** |  | **£95.67** |  | **£179.67** |  |
| Device and Insertion consumables | |  | | £802.47 |  | £697.00 |  | £877.47 | (27,28) |
| Diagnostic contrast fluoroscopy | |  | | £109 |  | £109.00 |  | £109.00 | (25) |
| Prophylactics antibiotics | |  | | £24.11 |  | £24.11 |  | £24.11 | (27,28) |
| Insertion X-ray | |  | | £31.30 |  | £31.30 |  | £31.30 | (27,28) |
| Insertion Renogram | |  | | N/A |  | N/A |  | £242.00 | (25) |
| CT scan | |  | | £79.78 |  | £79.78 |  | £79.78 | (25) |
| **TOTAL Equipment** | |  | | **£1,046.66** |  | **£941.19** |  | **£1,363.66** |  |
| **OVERALL TOTAL** | |  | | **£1,565.12** |  | **£1,316.73** |  | **£2,137.19** |  |

*^a^Band 5 or 6 anaesthetic assistant, therefore half time allocated to both, ^b^± Interquartile range calculated from IPD.*

|  | **JJ Insertion Base Case** | | | | | | | | | |
| --- | --- | --- | --- | --- | --- | --- | --- | --- | --- | --- |
|  |  | **Base case** | | | | **Lower** | | **Upper** | | **Sources** |
|  | **Hourly cost** | **Time required** | | | **Cost** | **Time required** | **Cost** | **Time required** | **Cost** |  |
| Anaesthetist | £123.00 | 20.6 minutes | | | £42.23 | 16.2 minutes | £33.21 | 25 minutes | £51.25 | (12) |
| Surgeon | £122.00 | 20.6 minutes | | | £41.89 | 16.2 minutes | £32.94 | 25 minutes | £50.83 | (12) |
| Registrar | £52.00 | 20.6 minutes | | | £17.85 | N/A | £0.00 | 25 minutes | £21.67 | (12) |
| Band 7 Scrub | £62.00 | 20.6 minutes | | | £21.29 | N/A | £0.00 | 25 minutes | £25.83 | (12) |
| Band 6 scrub | £51.00 | N/A | | | £0.00 | 16.2 minutes | £13.77 | 25 minutes | £21.25 | (12) |
| Band 5 scrub | £41.00 | 20.6 minutes | | | £14.08 | 16.2 minutes | £11.07 | N/A | £0.00 | (12) |
| Band 6 ODP^¥^ | £51.00 | 10.3 minutes | | | £8.76 | 8.1 minutes | £6.89 | 12.5 minutes | £10.63 | (12) |
| Band 5 ODP^¥^ | £41.00 | 10.3 minutes | | | £7.04 | 8.1 minutes | £5.54 | 12.5 minutes | £8.54 | (12) |
| Band 3 circulating | £26.50 | 20.6 minutes | | | £10.13 | 16.2 minutes | £7.97 | 25 minutes | £12.29 | (12) |
| Band 2 porters | £26.50 | 20.6 minutes | | | £9.10 | 16.2 minutes | £7.16 | 25 minutes | £11.04 | (12) |
| **TOTAL HCPs** | |  | | | **£175.70** |  | **£118.53** |  | **£213.33** |  |
| Theatre running cost | £637.00 | 20.6 minutes | | | £218.85 | 16.2 minutes | £172.10 | 25 minutes | £265.59 | (26) |
| **TOTAL theatre cost** | |  | | | **£391.20** |  | **£290.63** |  | **£478.92** |  |
| Band 6 recovery | £51.00 | 20 minutes | | | £17.00 | 20 minutes | £17.00 | 20 minutes | £17.00 | (27,28) |
| Band 5 recovery | £41.00 | 80 minutes | | | £54.67 | 80 minutes | £54.67 | 80 minutes | £54.67 | (27,28) |
| Bed stay | £288.00 | 4 hours | | £48.00 | | s hours | £24.00 | 9 hours | £108.00 | (27,28) |
| **RECOVERY TOTAL** | |  | **£119.67** | | |  | **£95.67** |  | **£179.67** |  |
| Device and Insertion consumables | |  | £116.14 | | |  | £58.64 |  | £308.47 | (27,28) |
| Diagnostic contrast fluoroscopy | |  | £109.00 | | |  | £109.00 |  | £109.00 | (25) |
| Prophylactics antibiotics | |  | £24.11 | | |  | £24.11 |  | £24.11 | (27,28) |
| Insertion X-ray | |  | £31.30 | | |  | £31.30 |  | £31.30 | (27,28) |
| Follow up outpatients | |  | N/A | | |  | N/A |  | £182.00 | (25) |
| CT scan | |  | £77.67 | | |  | £77.67 |  | £77.67 | (25) |
| **TOTAL Equipment** | |  | **£360.33** | | |  | **£302.84** |  | **£734.67** |  |
| **OVERALL TOTAL** | |  | **£871.20** | | |  | **£689.14** |  | **£1,393.26** |  |

*¥ Band 5 or 6 anaesthetic assistant, therefore half time allocated to both. *± 2 x SD*

#

|  | **Resonance Replacement Base Case** | | | | | | | | | |
| --- | --- | --- | --- | --- | --- | --- | --- | --- | --- | --- |
|  |  | **Base case** | | | | **Lower** | | **Upper** | | **Sources** |
|  | **Hourly cost** | **Time required** | | **Cost** | | **Time required** | **Cost** | **Time required** | **Cost** |  |
| Anaesthetist | £123.00 | 31 minutes | | £63.55 | | 25.6 minutes | £52.48 | 41 minutes | £84.05 | (15) ^b^ |
| Surgeon | £122.00 | 31 minutes | | £63.03 | | 25.6 minutes | £52.05 | 41 minutes | £83.37 | (15) ^b^ |
| Registrar | £52.00 | 31 minutes | | £26.87 | | N/A | £0.00 | 41 minutes | £35.53 | (15) ^b^ |
| Band 7 Scrub | £62.00 | 31 minutes | | £32.03 | | N/A | £0.00 | 41 minutes | £42.37 | (15) ^b^ |
| Band 6 scrub | £51.00 | N/A | | £0.00 | | 25.6 minutes | £21.76 | 41 minutes | £34.85 | (15) ^b^ |
| Band 5 scrub | £41.00 | 31 minutes | | £21.18 | | 25.6 minutes | £17.49 | N/A | £0.00 | (15) ^b^ |
| Band 6 ODP^a^ | £51.00 | 15.5 minutes | | £13.18 | | 12.8 minutes | £10.88 | 41 minutes | £17.43 | (15) ^b^ |
| Band 5 ODP^a^ | £41.00 | 15.5 minutes | | £10.59 | | 12.8 minutes | £8.75 | 20.5 minutes | £14.01 | (15) ^b^ |
| Band 3 circulating | £26.50 | 31 minutes | | £15.24 | | 25.6 minutes | £12.59 | 20.5 minutes | £20.16 | (15) ^b^ |
| Band 2 porters | £26.50 | 31 minutes | | £13.69 | | 25.6 minutes | £11.31 | 41 minutes | £18.11 | (15) ^b^ |
| **TOTAL HCPs** | |  | | **£259.37** | |  | **£187.31** |  | **£349.87** |  |
| Theatre running cost | £637.00 | 31 minutes | | £329.33 | | 25.6 minutes | £271.96 | 41 minutes | £435.57 | (26) |
| **TOTAL theatre cost** | |  | | **£588.70** | |  | **£459.43** |  | **£785.43** |  |
| Band 6 recovery | £51.00 | 20 minutes | | £17.00 | | 20 minutes | £17.00 | 20 minutes | £17.00 | (27,28) |
| Band 5 recovery | £41.00 | 80 minutes | | £54.67 | | 80 minutes | £54.67 | 80 minutes | £54.67 | (27,28) |
| Bed stay | £288.00 | 4 hours | | | £48.00 | 2 hours | £24.00 | 9 hours | £108.00 | (27,28) |
| **RECOVERY TOTAL** | |  | **£119.67** | | |  | **£95.67** |  | **£179.67** |  |
| Device and Insertion consumables | |  | £802.47 | | |  | £697.00 |  | £877.47 | (27,28) |
| Diagnostic contrast fluoroscopy | |  | £109 | | |  | £109.00 |  | £109.00 | (25) |
| Prophylactics antibiotics | |  | £24.11 | | |  | £24.11 |  | £24.11 | (27,28) |
| Insertion X-ray | |  | £31.30 | | |  | £31.30 |  | £31.30 | (27,28) |
| Insertion Renogram | |  | N/A | | |  | N/A |  | £242.00 | (25) |
| CT scan | |  | £77.67 | | |  | £77.67 |  | £77.67 | (25) |
| **TOTAL OTHER** | |  | **£1,046.66** | | |  | **£941.19** |  | **£1,363.66** |  |
| Oncology visit (stent fail) @ out patient cost | |  | £176.00 | | |  | £176.00 |  | £176.00 | (25) |
| Ambulance (stent fail) | |  | N/A | | |  | N/A |  | £265.00 | (25) |
| **Additional Stent failure costs** | |  | **£176.00** | | |  | **£176.00** |  | **£441.00** |  |
| **OVERALL TOTAL (planned)** | |  | **£1,755.02** | | |  | **£1,496.13** |  | **£2,328.76** |  |
| **OVERALL TOTAL (unplanned)** | |  | **£1,931.02** | | |  | **£1,672.13** |  | **£2,2,769.76** |  |

*^a^ Band 5 or 6 anaesthetic assistant, therefore half time allocated to both, ^b^± Interquartile range calculated from IPD.*

|  | **JJ Replacement Base Case** | | | | | | | | |
| --- | --- | --- | --- | --- | --- | --- | --- | --- | --- |
|  |  | **Base case** | | | **Lower** | | **Upper** | | **Sources** |
|  | **Hourly cost** | **Time required** | | **Cost** | **Time required** | **Cost** | **Time required** | **Cost** |  |
| Anaesthetist | £123.00 | 30.6 minutes | | £62.73 | 26.2 minutes | £53.71 | 35 minutes | £71.75 | (12) |
| Surgeon | £122.00 | 30.6 minutes | | £62.22 | 26.2 minutes | £53.27 | 35 minutes | £71.17 | (12) |
| Registrar | £52.00 | 30.6 minutes | | £26.52 | N/A | £0.00 | 35 minutes | £30.33 | (12) |
| Band 7 Scrub | £62.00 | 30.6 minutes | | £31.62 | N/A | £0.00 | 35 minutes | £36.17 | (12) |
| Band 6 scrub | £51.00 | N/A | | £0.00 | 26.2 minutes | £22.27 | 35 minutes | £29.75 | (12) |
| Band 5 scrub | £41.00 | 30.6 minutes | | £20.91 | 26.2 minutes | £17.90 | N/A | £0.00 | (12) |
| Band 6 ODP^a^ | £51.00 | 15.3 minutes | | £13.01 | 13.1 minutes | £11.14 | 17.5 minutes | £14.88 | (12) |
| Band 5 ODP^a^ | £41.00 | 15.3 minutes | | £10.46 | 13.1 minutes | £8.95 | 17.5 minutes | £11.96 | (12) |
| Band 3 circulating | £26.50 | 30.6 minutes | | £15.05 | 26.2 minutes | £12.88 | 35 minutes | £17.21 | (12) |
| Band 2 porters | £26.50 | 30.6 minutes | | £13.52 | 26.2 minutes | £11.57 | 35 minutes | £15.46 | (12) |
| **TOTAL HCPs** | |  | | **£256.02** |  | **£191.70** |  | **£298.67** |  |
| Theatre running cost | £637.00 | 30.6 minutes | | £325.08 | 26.2 minutes | £278.34 | 35 minutes | £371.83 | (26) |
| **TOTAL theatre cost** | |  | | **£581.10** |  | **£470.03** |  | **£670.49** |  |
| Band 6 recovery | £51.00 | 20 minutes | | £17.00 | 20 minutes | £17.00 | 20 minutes | £17.00 | (27,28) |
| Band 5 recovery | £41.00 | 80 minutes | | £54.67 | 80 minutes | £54.67 | 80 minutes | £54.67 | (27,28) |
| Bed stay | £288.00 | 4 hours | £48.00 | | 2 hours | £24.00 | 9 hours | £108.00 | (27,28) |
| **RECOVERY TOTAL** | |  | **£119.67** | |  | **£95.67** |  | **£179.67** |  |
| Device and Insertion consumables | |  | £115.06 | |  | £58.64 |  | £308.48 | (27,28) |
| Diagnostic contrast fluoroscopy | |  | £109.00 | |  | £109.00 |  | £109.00 | (25) |
| Prophylactics antibiotics | |  | £24.11 | |  | £24.11 |  | £24.11 | (27,28) |
| Insertion X-ray | |  | £31.30 | |  | £31.30 |  | £31.30 | (27,28) |
| Follow up outpatients | |  | N/A | |  | N/A |  | £182.00 | (25) |
| CT scan | |  | £77.67 | |  | £77.67 |  | £77.67 | (25) |
| **TOTAL Equipment** | |  | **£360.33** | |  | **£302.84** |  | **£734.67** |  |
| Oncology visit (stent fail) @ out patient cost | |  | £176.00 | |  | £176.00 |  | £176.00 | (25) |
| Ambulance (stent fail) | |  | N/A | |  | N/A |  | £265.00 | (25) |
| **Additional Stent failure costs** | |  | **£176.00** | |  | **£176.00** |  | **£441.00** |  |
| **OVERALL TOTAL (planned)** | |  | **£1,061.10** | |  | **£868.54** |  | **£1,584.83** |  |
| **OVERALL TOTAL (unplanned)** | |  | **£1,237.10** | |  | **£1.044.54** |  | **£2,025.83** |  |

*^a^ Band 5 or 6 anaesthetic assistant, therefore half time allocated to both.*

# Appendix 5 Scenario Analysis Data

|  | **Resonance Insertion Scenario Analysis** | | | | | | | | |
| --- | --- | --- | --- | --- | --- | --- | --- | --- | --- |
|  |  | **Base case** | | | **Lower** | | **Upper** | | **Sources** |
|  | **Hourly cost** | **Time required** | | **Cost** | **Time required** | **Cost** | **Time required** | **Cost** |  |
| Anaesthetist | £123.00 | 37.5 minutes | | £76.88 | 25 minutes | £51.25 | 55 minutes | £112.75 | (27,28) |
| Surgeon | £122.00 | 37.5 minutes | | £76.25 | 25 minutes | £50.83 | 55 minutes | £111.83 | (27,28) |
| Registrar | £52.00 | 37.5 minutes | | £32.50 | N/A | £0.00 | 55 minutes | £47.67 | (27,28) |
| Band 7 Scrub | £62.00 | 37.5 minutes | | £38.75 | N/A | £0.00 | 55 minutes | £56.83 | (27,28) |
| Band 6 scrub | £51.00 | N/A | | £0.00 | 25 minutes | £21.25 | 55 minutes | £46.75 | (27,28) |
| Band 5 scrub | £41.00 | 21 minutes | | £25.63 | 25 minutes | £17.08 | N/A | £0.00 | (27,28) |
| Band 6 ODP^a^ | £51.00 | 18.8 minutes | | £15.94 | 12.5 minutes | £10.63 | 27.5 minutes | £23.38 | (27,28) |
| Band 5 ODP^a^ | £41.00 | 18.8 minutes | | £12.81 | 12.5 minutes | £8.54 | 27.5 minutes | £18.79 | (27,28) |
| Band 3 circulating | £26.50 | 37.5 minutes | | £18.44 | 25 minutes | £12.29 | 55 minutes | £27.04 | (27,28) |
| Band 2 porters | £26.50 | 37.5 minutes | | £16.56 | 25 minutes | £11.04 | 55 minutes | £24.29 | (27,28) |
| **TOTAL HCPs** | |  | | **£313.75** |  | **£182.92** |  | **£469.33** |  |
| Theatre running cost | £624.00 | 37.5 minutes | | £398.39 | 25 minutes | £265.59 | 55 minutes | £584.59 | (26) |
| **TOTAL theatre cost** | |  | | **£712.14** |  | **£448.51** |  | **£1,053.63** |  |
| Band 6 recovery | £51.00 | 20 minutes | | £17.00 | 20 minutes | £17.00 | 20 minutes | £17.00 | (27,28) |
| Band 5 recovery | £41.00 | 80 minutes | | £54.67 | 80 minutes | £54.67 | 80 minutes | £54.67 | (27,28) |
| Bed stay | £288.00 | 4 hours | £48.00 | | 2 hours | £24.00 | 24 hours | £288.00 | (27,28) |
| **RECOVERY TOTAL** | |  | **£119.67** | |  | **£95.67** |  | **£359.67** |  |
| Device and Insertion consumables | |  | £802.47 | |  | £697.00 |  | £877.47 | (27,28) |
| Diagnostic contrast fluoroscopy | |  | £109.00 | |  | £109.00 |  | £109.00 | (25) |
| Prophylactics antibiotics | |  | £24.11 | |  | £24.11 |  | £24.11 | (27,28) |
| Insertion X-ray | |  | £31.30 | |  | £31.30 |  | £31.30 | (27,28) |
| Insertion Renogram | |  | £242.00 | |  | N/A |  | £242.00 | (25) |
| CT scan | |  | £77.67 | |  | £77.67 |  | £77.67 | (25) |
| **TOTAL Equipment** | |  | **£1,288.66** | |  | **£941.19** |  | **£1,363.66** |  |
| **OVERALL TOTAL** | |  | **£2,120.46** | |  | **£1,485.36** |  | **£2,776.96** |  |

*^a^ Band 5 or 6 anaesthetic assistant, therefore half time allocated to both, NHSSC, NHS Supply Chain Catalogue*

|  | **Polyurethane J J Insertion Scenario Analysis** | | | | | | | |
| --- | --- | --- | --- | --- | --- | --- | --- | --- |
|  |  | **Base case** | | **Lower** | | **Upper** | | **Sources** |
|  | **Hourly cost** | **Time required** | **Cost** | **Time required** | **Cost** | **Time required** | **Cost** |  |
| Anaesthetist | £123.00 | 22.5 minutes | £46.13 | 15 minutes | £30.75 | 35 minutes | £71.75 | (27,28) |
| Surgeon | £122.00 | 22.5 minutes | £45.75 | 15 minutes | £30.50 | 35 minutes | £71.17 | (27,28) |
| Registrar | £52.00 | 22.5 minutes | £19.50 | N/A | £0.00 | 35 minutes | £30.33 | (27,28) |
| Band 7 Scrub | £62.00 | 22.5 minutes | £23.25 | N/A | £0.00 | 35 minutes | £36.17 | (27,28) |
| Band 6 scrub | £51.00 | N/A | £0.00 | 15 minutes | £12.75 | 35 minutes | £29.75 | (27,28) |
| Band 5 scrub | £41.00 | 22.5 minutes | £15.38 | 15 minutes | £10.25 | N/A | £0.00 | (27,28) |
| Band 6 ODP^a^ | £51.00 | 11.25 minutes | £9.56 | 7.5 minutes | £6.38 | 17.5 minutes | £14.88 | (27,28) |
| Band 5 ODP^a^ | £41.00 | 11.25 minutes | £7.69 | 7.5 minutes | £5.13 | 17.5 minutes | £11.96 | (27,28) |
| Band 3 circulating | £26.50 | 22.5 minutes | £11.06 | 15 minutes | £7.38 | 35 minutes | £17.21 | (27,28) |
| Band 2 porters | £26.50 | 22.5 minutes | £9.94 | 15 minutes | £6.63 | 35 minutes | £15.46 | (27,28) |
| **TOTAL HCPs** | |  | **£188.25** |  | **£109.75** |  | **£298.67** |  |
| Theatre running cost | £624.00 | 22.5 minutes | £239.00 | 15 minutes | £159.35 | 35 minutes | £371.83 | (26) |
| **TOTAL theatre cost** | |  | **£427.28** |  | **£269.10** |  | **£670.49** |  |
| Band 6 recovery | £51.00 | 20 minutes | £17.00 | 20 minutes | £17.00 | 20 minutes | (27,28) | (27,28) |
| Band 5 recovery | £41.00 | 80 minutes | £54.67 | 80 minutes | £54.67 | 80 minutes | (27,28) | (27,28) |
| Bed stay | £288.00 | 4 hours | £48.00 | 2 hours | £24.00 | 24 hours | (27,28) | (27,28) |
| **RECOVERY TOTAL** | |  | **£119.67** |  | **£95.67** |  | **£259.67** |  |
| Device and Insertion consumables | |  | £116.14 |  | £58.64 |  | £308.47 | (27,28) |
| Diagnostic contrast fluoroscopy | |  | £109.00 |  | £109.00 |  | £109.00 | (25) |
| Prophylactics antibiotics | |  | £24.11 |  | £24.11 |  | £24.11 | (27,28) |
| Insertion X-ray | |  | £31.30 |  | £31.30 |  | £31.30 | (27,28) |
| Follow up outpatients | |  | £182.00 |  | N/A |  | £182.00 | (25) |
| CT scan | |  | £77.67 |  | £77.67 |  | £77.67 | (25) |
| **TOTAL Equipment** | |  | **£542.33** |  | **£302.84** |  | **£734.67** |  |
| **OVERALL TOTAL** | |  | **£1,089.28** |  | **£667.61** |  | **£1,764.83** |  |

*^a^ Band 5 or 6 anaesthetic assistant, therefore half time allocated to both.*

## Scenario Analysis. Replacement Costs and Data for Sensitivity Analysis

|  | **Resonance Replacement Scenario Analysis** | | | | | | | | |
| --- | --- | --- | --- | --- | --- | --- | --- | --- | --- |
|  |  | **Base case** | | | **Lower** | | **Upper** | | **Sources** |
|  | **Hourly cost** | **Time required** | | **Cost** | **Time required** | **Cost** | **Time required** | **Cost** |  |
| Anaesthetist | £123.00 | 67.5 minutes | | £138.38 | 30 minutes | £61.50 | 90 minutes | (27,28) | (27,28) |
| Surgeon | £122.00 | 67.5 minutes | | £137.25 | 30 minutes | £61.00 | 90 minutes | (27,28) | (27,28) |
| Registrar | £52.00 | 67.5 minutes | | £58.50 | N/A | £0.00 | 90 minutes | (27,28) | (27,28) |
| Band 7 Scrub | £62.00 | 67.5 minutes | | £69.75 | N/A | £0.00 | 90 minutes | (27,28) | (27,28) |
| Band 6 scrub | £51.00 | N/A | | £0.00 | 30 minutes | £25.50 | 90 minutes | (27,28) | (27,28) |
| Band 5 scrub | £41.00 | 67.5 minutes | | £46.13 | 30 minutes | £20.50 | N/A | (27,28) | (27,28) |
| Band 6 ODP^a^ | £51.00 | 33.75 minutes | | £28.69 | 15 minutes | £12.75 | 90 minutes | (27,28) | (27,28) |
| Band 5 ODP^a^ | £41.00 | 33.75 minutes | | £23.06 | 15 minutes | £10.25 | 45 minutes | (27,28) | (27,28) |
| Band 3 circulating | £26.50 | 67.5 minutes | | £33.19 | 30 minutes | £14.75 | 45 minutes | (27,28) | (27,28) |
| Band 2 porters | £26.50 | 67.5 minutes | | £29.81 | 30 minutes | £13.25 | 90 minutes | (27,28) | (27,28) |
| **TOTAL HCPs** | |  | | **£564.75** |  | **£219.50** |  | **£768.00** |  |
| Theatre running cost | £624.00 | 67.5 minutes | | £717.09 | 30 minutes | £318.71 | 90 minutes | £956.12 | (26) |
| **TOTAL theatre cost** | |  | | **£1,266.75** |  | **£531.50** |  | **£1,704.00** |  |
| Band 6 recovery | £51.00 | 20 minutes | | £17.00 | 20 minutes | £17.00 | 20 minutes | (27,28) | (27,28) |
| Band 5 recovery | £41.00 | 80 minutes | | £54.67 | 80 minutes | £54.67 | 80 minutes | (27,28) | (27,28) |
| Bed stay | £288.00 | 4 hours | £48.00 | | 2 hours | £24.00 | 24 hours | (27,28) | (27,28) |
| **RECOVERY TOTAL** | |  | **£119.67** | |  | **£95.67** |  | **£359.67** |  |
| Device and Insertion consumables | |  | £802.47 | |  | £697.00 |  | £877.47 | (27,28) |
| Diagnostic contrast fluoroscopy | |  | £109.00 | |  | £109.00 |  | £109.00 | (25) |
| Prophylactics antibiotics | |  | £24.11 | |  | £24.11 |  | £24.11 | (27,28) |
| Insertion X-ray | |  | £31.30 | |  | £31.30 |  | £31.30 | (27,28) |
| Insertion Renogram | |  | £242.00 | |  | N/A |  | £242.00 | (25) |
| CT scan | |  | £77.67 | |  | £77.67 |  | £77.67 | (25) |
| **TOTAL OTHER** | |  | **£1,288.66** | |  | **£941.19** |  | **£1,363.66** |  |
| Oncology visit (stent fail) @ out patient cost | |  | £176.00 | |  | £176.00 |  | £176.00 | (25) |
| Ambulance (stent fail) | |  | N/A | |  | N/A |  | £265.00 | (25) |
| **Additional Stent failure costs** | |  | **£176.00** | |  | **£176.00** |  | **£441.00** | (27,28) |
| **OVERALL TOTAL (planned)** | |  | **£2,690.17** | |  | **£1,575.07** |  | **£3,447.45** | (27,28) |
| **OVERALL TOTAL (unplanned)** | |  | **£2,866.17** | |  | **£1,751.07** |  | **£3,888.45** | (27,28) |

*^a^ Band 5 or 6 anaesthetic assistant, therefore half time allocated to both.*

|  | **Polyurethane Replacement Scenario Analysis** | | | | | | | | |
| --- | --- | --- | --- | --- | --- | --- | --- | --- | --- |
|  |  | **Base case** | | | **Lower** | | **Upper** | | **Sources** |
|  | **Hourly cost** | **Time required** | | **Cost** | **Time required** | **Cost** | **Time required** | **Cost** |  |
| Anaesthetist | £123.00 | 52.5 minutes | | £107.63 | 30 minutes | £61.50 | 80 minutes | (27,28) | (27,28) |
| Surgeon | £122.00 | 52.5 minutes | | £106.75 | 30 minutes | £61.00 | 80 minutes | (27,28) | (27,28) |
| Registrar | £52.00 | 52.5 minutes | | £45.50 | N/A | £0.00 | 80 minutes | (27,28) | (27,28) |
| Band 7 Scrub | £62.00 | 52.5 minutes | | £54.25 | N/A | £0.00 | 80 minutes | (27,28) | (27,28) |
| Band 6 scrub | £51.00 | N/A | | £0.00 | 30 minutes | £25.50 | 80 minutes | (27,28) | (27,28) |
| Band 5 scrub | £41.00 | 52.5 minutes | | £35.88 | 30 minutes | £20.50 | N/A | (27,28) | (27,28) |
| Band 6 ODP^¥^ | £51.00 | 26.25 minutes | | £22.31 | 15 minutes | £12.75 | 40 minutes | (27,28) | (27,28) |
| Band 5 ODP^¥^ | £41.00 | 26.25 minutes | | £17.94 | 15 minutes | £10.25 | 40 minutes | (27,28) | (27,28) |
| Band 3 circulating | £26.50 | 52.5 minutes | | £25.81 | 30 minutes | £14.75 | 80 minutes | (27,28) | (27,28) |
| Band 2 porters | £26.50 | 52.5 minutes | | £23.19 | 30 minutes | £13.25 | 80 minutes | (27,28) | (27,28) |
| **TOTAL HCPs** | |  | | **£439.25** |  | **£219.50** |  | **£682.67** |  |
| Theatre running cost | £624.00 | 52.5 minutes | | £557.74 | 26.2 minutes | £318.71 | 35 minutes | £849.89 | (26) |
| **TOTAL theatre cost** | |  | | **£996.99** |  | **£538.21** |  | **£1,532.55** |  |
| Band 6 recovery | £51.00 | 20 minutes | | £17.00 | 20 minutes | £17.00 | 20 minutes | (27,28) | (27,28) |
| Band 5 recovery | £41.00 | 80 minutes | | £54.67 | 80 minutes | £54.67 | 80 minutes | (27,28) | (27,28) |
| Bed stay | £288.00 | 4 hours | | £48.00 | 2 hours | £24.00 | 24 hours | (27,28) | (27,28) |
| **RECOVERY TOTAL** | |  | **£119.67** | |  | **£95.67** |  | **£359.67** |  |
| Device and Insertion consumables | |  | £116.14 | |  | £58.64 |  | £308.48 | (27,28) |
| Diagnostic contrast fluoroscopy | |  | £109.00 | |  | £109.00 |  | £109.00 | (25) |
| Prophylactics antibiotics | |  | £24.11 | |  | £24.11 |  | £24.11 | (27,28) |
| Insertion X-ray | |  | £31.30 | |  | £31.30 |  | £31.30 | (27,28) |
| Follow up outpatients | |  | £182.00 | |  | N/A |  | £182.00 | (25) |
| CT scan | |  | £77.67 | |  | £77.67 |  | £77.67 | (25) |
| **TOTAL Equipment** | |  | **£542.33** | |  | **£302.84** |  | **£734.67** |  |
| Oncology visit (stent fail) @ out patient cost | |  | £176.00 | |  | £176.00 |  | £176.00. | (25) |
| Ambulance (stent fail) | |  | N/A | |  | N/A |  | £265.00 | (25) |
| **Additional Stent failure costs** | |  | **£176.00** | |  | **£176.00** |  | **£441.00** | (27,28) |
| **OVERALL TOTAL (planned)** | |  | **£1,658.99** | |  | **£936.71** |  | **£2,626.89** | (27,28) |
| **OVERALL TOTAL (unplanned)** | |  | **£1,834.99** | |  | **£1,112.71** |  | **£3,067.89** | (27,28) |

*¥ Band 5 or 6 anaesthetic assistant, therefore half time allocated to both. *± 2 x SD*

## Scenario Analysis Cost-Effectiveness Plane

## Scenario Analysis, Cost-Effectiveness Acceptability Curve

## Scenario Analysis, DSA Incremental costs

## Scenario Analysis, DSA Incremental NMB
