# Supplementary material for: Cost‐effectiveness of Resonance® metallic ureteral stent compared with standard polyurethane ureteral stents in malignant ureteric obstruction: A cost‐utility analysis
Source: BJUI Compass. 2024 Mar 20;5(5):465–75. doi: 10.1002/bco2.332 (PMC11090770; doi:10.1002/bco2.332)
Supplement: Supplementary file 2 — Data S1. Supporting Information. [file BCO2-5-465-s002.pdf]

Consolidated Health Economic Evaluation Reporting Standards (CHEERS) 2022 Checklist

The CHEERS 2022 statement replaces the 2013 CHEERS statement, which should no longer be used. The CHEERS 2022 checklist contains 28 items with accompanying descriptions. Checklist users should indicate the section of the manuscript where relevant information can be found. The authors recommend using a section heading with a paragraph number. If an item does not apply to a particular economic evaluation, checklist users are encouraged to report "Not Applicable." If information is otherwise not reported, checklist users are encouraged to write, "Not Reported." Users should avoid the term "Not Conducted" as CHEERS is intended to guide and capture reporting. Additional information on CHEERS 2022 can be found here.

Title

1. Title

Identify the study as an economic evaluation and specify the interventions being compared.

Yes, Title, Page 1

Abstract

2. Abstract

Provide a structured summary that highlights context, key methods, results, and alternative analyses.

Yes, Abstract page 2.

Introduction

3. Introduction: Background and Objectives

Give the context for the study, the study question, and its practical relevance for decision making in policy or practice.

Context and relevance for decision making in section 1 "Introduction" on page 2-3
Objectives, section 1.1 "Objectives" on page 3.

Methods

4. Health economic analysis plan

© 2022 ISPOR—The Professional Society for Health Economics and Outcomes Research
International Society for Pharmacoeconomics and Outcomes Research, Inc

Indicate whether a health economic analysis plan was developed and where available.

Not applicable.

5. Study population

Describe characteristics of the study population (such as age range, demographics, socioeconomic, or clinical characteristics).

2.1 methods page 3, paragraph 4, lines 8-10.

6. Setting and location

Provide relevant contextual information that may influence findings.

2.1 methods page 3, paragraph 4, line 3

7. Comparators

Describe the interventions or strategies being compared and why chosen.

2.1 methods page 3, paragraph 4, lines1-3 and section 1 Introduction page 3 paragraphs 1 an 2.

8. Perspective

State the perspective(s) adopted by the study and why chosen.

2.1 methods page 3, paragraph 4, line 3

9. Time horizon

State the time horizon for the study and why appropriate.

2.1 methods page 3, paragraph 4, line 5

10. Discount rate

Report the discount rate(s) and reason chosen.

2.1 methods page 3, paragraph 4, line 3

11. Selection of outcomes

Describe what outcomes were used as the measure(s) of benefit(s) and harm(s).

2.1 methods page 3, paragraph 4, lines 6-15

12. Measurement of outcomes

Describe how outcomes used to capture benefit(s) and harm(s) were measured.

Section 2.3 clinial inputs page 5-6

© 2022 ISPOR—The Professional Society for Health Economics and Outcomes Research

International Society for Pharmacoeconomics and Outcomes Research, Inc

13. Valuation of outcomes

Describe the population and methods used to measure and value outcomes.

Section 2.3 clinial inputs page 5-6

14. Measurement and valuation of resources and costs

Describe how costs were valued.

Section 2.4 costs page 7

15. Currency, price date, and conversion

Report the dates of the estimated resource quantities and unit costs, plus the currency and year of conversion.

Page 6 table 2 and Appendix 2

16. Rationale and description of model

If modeling is used, describe in detail and why used. Report if the model is publicly available and where it can be accessed.

Section 2 1 (page 3) and figure 1.

17. Analytics and assumptions

Describe any methods for analyzing or statistically transforming data, any extrapolation methods, and approaches for validating any model used.

Appendix 3

18. Characterizing heterogeneity

Describe any methods used for estimating how the results of the study vary for subgroups.

Not applicable

19. Characterizing distributional effects

Describe how impacts are distributed across different individuals or adjustments made to reflect priority populations.

Not applicable

20. Characterizing uncertainty

Describe methods to characterize any sources of uncertainty in the analysis.

Section 2.6 Sensitivity analysis and Section 2.7 scenario analysis. Details in Appendix 4

21.

21. Approach to engagement with patients and others affected by the study

© 2022 ISPOR—The Professional Society for Health Economics and Outcomes Research

International Society for Pharmacoeconomics and Outcomes Research, Inc

Describe any approaches to engage patients or service recipients, the general public, communities, or stakeholders (eg, clinicians or payers) in the design of the study.

Not applicabel -co-authors are clinicians.

Results

22. Study parameters

Report all analytic inputs (eg, values, ranges, references) including uncertainty or distributional assumptions.

Not applicabel -co-authors are clinicians.

23. Summary of main results

Report the mean values for the main categories of costs and outcomes of interest and summarize them in the most appropriate overall measure.

Table 2 page 6 and appendix 2

24. Effect of uncertainty

Describe how uncertainty about analytic judgments, inputs, or projections affects findings. Report the effect of choice of discount rate and time horizon, if applicable.

Table 3 page 9, Section 3.2 Sensitivity analysis and section 3 scenario analysis on page 9 and appendix 5

25. Effect of engagement with patients and others affected by the study

Report on any difference patient/service recipient, general public, community, or stakeholder involvement made to the approach or findings of the study.

Not applicable

Discussion

26. Study findings, limitations, generalizability, and current knowledge

Report key findings, limitations, ethical, or equity considerations not captured and how these could impact patients, policy, or practice.

Section 4 discussion pages 11-13

© 2022 ISPOR—The Professional Society for Health Economics and Outcomes Research
International Society for Pharmacoeconomics and Outcomes Research, Inc

Other Relevant Information

27. Source of funding

Describe how the study was funded and any role of the funder in the identification, design, conduct, and reporting of the analysis.

Not applicable

28. Conflicts of interest

Report authors' conflicts of interest according to journal or International Committee of Medical Journal Editors requirements.

Page 14 "conflicts of interest"

© 2022 ISPOR—The Professional Society for Health Economics and Outcomes Research
International Society for Pharmacoeconomics and Outcomes Research, Inc
